# The Role of NADPH Oxidase 4 in Neutrophil-Mediated Immune Escape in Gastric Cancer

**DOI:** 10.7150/ijbs.121960

**Published:** 2026-05-01

**Authors:** Jinling Jiang, Wenqi Xi, Yanan Zheng, Jun Ji, Chenfei Zhou, Min Shi, Junwei Wu, Yangbing Jin, Benyan Zhang, Liqin Zhao, Qu Cai, Jun Zhang

**Affiliations:** 1 Department of Oncology, Ruijin Hospital, Shanghai Jiao Tong University School of Medicine, No. 197 Ruijin er Road, Shanghai, 200025, China.; 2 Shanghai Key Laboratory of Gastric Neoplasms, No. 197 Ruijin er Road, Shanghai, 200025, China.; 3 Department of Surgery, Ruijin Hospital, Shanghai Jiao Tong University School of Medicine, No. 197 Ruijin er Road, Shanghai, 200025, China.; 4 Shanghai Institute of Digestive Surgery, Ruijin Hospital, Shanghai Jiao Tong University School of Medicine, No. 197 Ruijin er Road, Shanghai, 200025, China.; 5 Department of Pathology, Ruijin Hospital, Shanghai Jiao Tong University School of Medicine, No. 197 Ruijin er Road, Shanghai, 200025, China.

**Keywords:** gastric cancer, NADPH oxidase 4, neutrophil activation, immune suppression

## Abstract

NADPH oxidase 4 (NOX4) transmits electrons for various redox reactions by generating reactive oxygen species (ROS), which are present at high levels in a variety of malignant tumors, including gastric cancer (GC). Nevertheless, the role of NOX4 in inducing immune escape in GC remains unknown. We analyzed the correlation between NOX4 expression and infiltrating neutrophils in human GC tissue. NOX4 overexpression correlated with poor prognosis and enhanced neutrophil infiltration in GC patients and mouse models. Tumor-derived NOX4 promoted secretion of GM-CSF, which was established through *in vivo* functional assays as the key factor responsible for neutrophil recruitment. Recruited neutrophils inhibited GC cell apoptosis and fostered an immunosuppressive microenvironment. NOX4/GM-CSF signaling further enhanced PHGDH and ASNS-mediated metabolic adaptation in neutrophils. Elevated NOX4 and neutrophil infiltration were observed in GC patients unresponsive to neoadjuvant anti-PD-1 therapy. Tumor-derived NOX4 drives GM-CSF-dependent neutrophil recruitment, leading to metabolic reprogramming and immunosuppression in GC. Targeting the GM-CSF/neutrophil axis may overcome resistance to immune checkpoint inhibitors.

## 1. Introduction

Gastric cancer (GC) is one of the most heterogeneous types of cancer 1. Complex molecular and phenotypic heterogeneity contributes to the limited benefit from the "one size fits all" treatment strategy, resulting in a poor prognosis, with a 5-year survival of less than 30% 2. GC pathogenesis has not been fully elucidated. The occurrence, development, and prognosis of GC are closely related to the interactions between tumor cells and immune cells in the microenvironment 3, 4.

The tumor microenvironment is characterized by a higher level of reactive oxygen species (ROS) than other microenvironments 5. Cancer cells produce ROS primarily from nicotinamide adenine dinucleotide phosphate (NADPH) oxidases (NOXs) 6. NOX4, one of the NOX family members, has been reported to be highly expressed in various digestive tract malignancies, including GC, and is negatively correlated with prognosis 7-9. In addition, previous studies have revealed that NOX4 interacts with different immune cells within the tumor microenvironment, including tumor-associated macrophages (TAMs) 10, natural killer (NK) cells 11 and T cells 12, to synergistically promote tumor progression. Regardless, the mechanisms underlying NOX4-mediated GC malignant progression are only just been revealed.

With the advent of the immune era, accumulating evidence supports the notion that tumor progression and immunotherapy are influenced by immune cells in the tumor microenvironment 13. Neutrophils, the most abundant leukocytes, are the most common infiltrating immune cells in the GC microenvironment. Similar to TAMs, neutrophils are polarized into either antitumor N1 or protumor N2 cells 14. Recent studies have shown that tumor-infiltrating neutrophils overexpress PD-L1 or PD-L2 as a result of the regulation of intracellular signaling pathways and can inhibit T-cell function through binding with PD-1 or PD-2 on T cells to exert an immunosuppressive effect, which is negatively correlated with the prognosis of GC patients 15, 16. These results suggest that neutrophils might be a therapeutic target for GC. Notably, the correlation between NOX4 and neutrophils has been largely studied in nontumor diseases, such as acute lung injury 17, sepsis 18, and neutrophilic asthma 19. However, virtually nothing is known about how NOX4 regulates neutrophil recruitment, polarization, and protumor functions in malignant tumors.

In the current study, we showed that high NOX4 expression adversely affects GC patient prognosis and increases neutrophil recruitment and activity in GC patients and mice. Moreover, we observed that NOX4 overexpression in GC cells prolonged the neutrophil lifespan and affected neutrophil polarity. In turn, activated neutrophils significantly inhibited GC cell apoptosis. Mechanistically, we demonstrated that tumor-derived NOX4 recruits neutrophils to tumor sites by secreting granulocyte-macrophage colony-stimulating factor (GM-CSF), which causes neutrophil metabolic remodeling. Furthermore, in our cohort, the presence of higher NOX4 expression and neutrophil infiltration was associated with reduced efficacy of neoadjuvant anti-PD-1 therapy (camrelizumab) in GC patients.

## 2. Methods

### 2.1. Reagents

The NOX4 inhibitor GKT137831 (S7171), the ROS scavager acetylcysteine (NAC S1632), Bevacizumab (S2006), the inhibitor PHGDH (CBR-5884, S9645), the inhibitor PLA2G3 (Trans-4-Phenyl-3-buten-2-one, S9478) were purchased from Selleckchem. The Calcein AM (C2012) was purchased from Beyotime Biotechnology. The human CD14 neutralizing antibody (MAB247), the human GM-CSF neutralizing antibody (MAB215), the human IL-15 neutralizing antibody (MAB247) and human DKK-1 neutralizing antibody (MAB1096) were purchased from R&D. The inhibitor ASNS (L-asparaginase, ZY9015) was purchased from Merk. The human inhibitor PD-1(carelizumab, S20190027) was friendship provided by Jiangsu *Hengrui Pharmaceutical*s *Co.,Ltd.*

### 2.2. Specimens

Tumor and peritumor tissues and peripheral blood of patients with GC who underwent primary tumor resection at Ruijin Hospital Affiliated to Shanghai Jiao Tong University School of Medicine (China) between May 2015 and September 2018 were collected. We obtained snap-frozen GC tissues from patients who underwent curative resection at Ruijin Hospital between June 2018 and December 2018 and used them for quantitative real-time polymerase chain reaction (qRT‒PCR) and Western blot (Wb) analyses. All patients signed informed consent forms before surgery, and no chemotherapy, radiotherapy or immunotherapy had been administered to them prior to surgery. In addition, tissue specimens from 16 patients who received neoadjuvant immunotherapy with the anti-PD-1 agent carelizumab (200 mg single dose) were collected from a clinical trial (NCT04208347) performed at Ruijin Hospital.

Patients with autoimmune disease or infectious disease, who had received white blood cell-elevating drug therapy or who had more than one primary cancer were excluded. We performed *in vitro* and *in vivo* functional experiments with neutrophils obtained from the peripheral blood of 20 healthy donors. The inclusion criteria were as follows: Age 18-75 years old; No underlying diseases that can cause neutrophil elevation, such as chronic infections, autoimmune diseases, leukemia, etc.; No other chronic basic diseases, such as hypertension, diabetes, chronic kidney disease, etc.; No history of benign or malignant tumors.

### 2.3. Cell culture and materials

American Type Culture Collection (ATCC, Manassas, VA, USA) provided human GC cells (NCI-N87 and AGS, RRID: CVCL_1603 and RRID: CVCL_0139, respectively). The Chinese Academy of Science provided BGC-823 (RRID: CVCL_3360), SNU-1 (RRID: CVCL_0099), SNU-16 (RRID: CVCL_0076), KATO III (RRID: CVCL_0371), MKN-45 (RRID: CVCL_0434), SGC-7901 (RRID: CVCL_0520), MGC-803 (RRID: CVCL_5334) and normal gastric mucosal GES-1 cells (RRID: CVCL_EQ22). Human umbilical vein endothelial cells (HUVECs, RRID: CVCL_2959) were preserved and passaged at the Shanghai Institute of Digestive Surgery. All experimental cells were cultured in DMEM supplemented with 10% FBS, 100 U/ml penicillin G and 100μg/ml streptomycin. The incubator was maintained at 37°C in 5% CO_2_. Short tandem repeat (STR) profiling was used to confirm that the cell lines used were mycoplasma-free.

### 2.4. Bioinformatics analysis

The mRNA expression profile data used in this study were downloaded from Gene Expression Profiling Interactive Analysis (GEPIA, http://gepia.cancer-pku.cn/). Survival analysis was performed on genes differentially expressed between NOX4^low^ and NOX4^high^ groups using the Kaplan‒Meier Plotter database (https://kmplot.com/analysis).

### 2.5. GC cells, plasmids, lentivirus production, and cell transduction

In this study, MKN45 and NCI-N87 cells were used for NOX4 overexpression and/or knockdown experiments. The MKN45 cell line, established from a liver metastasis of gastric cancer, was selected for its aggressive characteristics and relevance to studying advanced GC phenotypes. The NCI-N87 gastric carcinoma cell line was used in parallel to ensure the generalizability of the findings. GeneChem (Shanghai, China) provided the LVNOX4 (44903) plasmids and NOX4 lentiviral shRNA. The shRNA sequences for NOX4 used in this study are as follows: shRNA1: AGAGTATCAC TACCTCCACCAGATGTTGG (sense), shRNA2: AACCTCTTCTTTGTCTTCT ACATGCTGCT (sense), and shRNA3: TCCTGGGCTTCATTGACATCTTCAA CTCA. As directed by the manufacturer, NOX4 plasmids or lentiviruses were transfected into the cells. NOX4 over expression cells were defined as NOX4-OE and NOX4 under expression as NOX4-KD. Wb, ROS and qRT‒PCR assays were used to measure intervention efficiency.

### 2.6. Extraction of RNA and qRT‒PCR

TRIzol (Invitrogen) was used to extract total RNA, and Superscript III reverse transcriptase (Invitrogen) was used to reverse transcribing the RNA. Based on the PrimeScriptTMRT Reagent Kit with gDNA Eraser (Perfect Real Time) protocols, random primers were used. qRT- PCR was performed using SYBR Green premix Ex Taq on the ABI ViiA 7 Dx RT-PCR system. Human β-actin served as an internal reference. The 2^-ΔΔCt^ method was used to calculate relative mRNA expression. Supplementary Table 1 provides the primer sequences.

### 2.7. Wb

We lysed cultured or human tissue cells in RIPA lysis buffer (Sigma-Aldrich) that contained protease and phosphatase inhibitors (Sigma-Aldrich). Wet blotting (BIO-RAD) was used to transfer proteins from 10% SDS-PAGE gels to PVDF membranes. After being blocked in 5% BSA in TBST and overnight incubation at 4°C, the membranes were incubated for 1-2 hours with species-specific secondary antibodies at room temperature. For protein band detection, chemiluminescence was measured using a Tanon-4200 (LI-COR Biosciences). Supplementary Table 2 lists the antibodies used.

### 2.8. ROS assay

H2O2 was measured using the Amplex Red® hydrogen peroxide assay kit (A22188, Invitrogen) in different NOX4 expression GC cells. Horseradish peroxidase (HRP) was used as a substrate to detect cellular H2O2 production using 10-acetyl-3, 7 dihydroxyphenoxazine. Resorufin is formed when this reagent reacts with H2O2 in the presence of peroxidase. The fluorescence of resorufin (excitation at 530 nm and emission at 590 nm) was detected in a plate reader at 37 °C.

### 2.9. Animal models

The Shanghai Laboratory Animal Center of the Chinese Academy of Science provided nonobese diabetic severe combined immunodeficiency (NOD-SCID) mice and BALB/c nude mice for this experiment. Those mice were bred under specific pathogen-free conditions. Shanghai Medical Experimental Animal Care Committee guidelines were strictly followed in all mouse experiments and National Academy of Sciences and National Institutes of Health guidelines were followed. The maximum permitted tumor burden was set at 10% of the initial body weight (approximately 2.2 grams), with a maximum diameter not exceeding 20 mm in any direction. To ensure strict compliance, any mouse that approached or reached either of these limits, or showed any signs of distress, was humanely euthanized immediately without exception.

2~3×10^6^ cells/100 μL of MKN45, MKN45-NOX4-OE, MKN45-NOX4-KD, NCI -N87 and NCI-N87-NOX4-KD cells were injected subcutaneously into the right flank. The tumor sizes were calculated with the following formula: (mm^3^) = (L×W^2^) ×0.5. We measured the tumor volume every four days.

To evaluate the *in vivo* chemotaxis of neutrophils toward GC tumors, we established subcutaneous xenograft models by inoculating MKN45 and MKN45-NOX4-OE cells (2×10⁶ cells in 100μL) into 6-8-week-old NOD/SCID mice. When the tumors reached approximately 2 mm³, we intravenously injected human neutrophils (5×10⁶ cells in 200μL) via the tail vein. These neutrophils were isolated from healthy donors and pre-activated for 24 hour by incubation with conditioned medium from MKN45-NOX4-OE cells. To investigate the roles of specific chemokines, neutrophils were pre-treated for 1 hour with neutralizing antibodies against CD14, GM-CSF, or DKK-1 (20μg/mL) prior to injection. Tumor dimensions were measured regularly to calculate volumes, and the mice were sacrificed eight weeks post-inoculation. Subsequently, tumor specimens were collected and processed for IHC staining of CD66b (a neutrophil marker) and TUNEL staining (to assess apoptosis).

The NOD/SCID mouse model was constructed following the same protocols as above chemotactic experiments. We induced activation of neutrophils and pretreated them for 24 hours with inhibitor of PHGDH (used at 15μM), inhibitor of ASNS (used at 20μM) and inhibitor of PLA2G3 (used at 50μM) following injection into mice's tail veins. After 8 weeks, the tumor size was detected, and IHC to detect CD66b and TUNEL staining were performed.

Human neutrophils isolated from healthy donors were pre-activated for 24 hours with conditioned medium from NOX4-overexpressing MKN45 cells. Gastric cancer cells (MKN45 or NCI-N87, 1 × 10⁶) were then co-injected with either activated or resting neutrophils (5 × 10⁶) via tail vein into NOD/SCID mice to establish a lung metastasis model. For the inhibitor group, mice received daily intraperitoneal injections of GKT137831 (30 mg/kg/day) starting one day before cell injection and continuing for 6 weeks. After 6 weeks, mice were euthanized and lung metastatic nodules were counted. Lung tissues were further processed for H&E staining and immunohistochemical analysis.

### 2.10. IHC

Clinical samples from 203 GC and adjacent tissues were acquired at Ruijin Hospital Affiliated to Shanghai Jiaotong University School of Medicine. Tissues were formalin-fixed, paraffin-embedded, and analyzed by IHC using the indicated antibodies: anti-NOX4 and anti-CD66b. Detailed information on the antibodies used can be found in Supplementary Table 2. Images were captured using an Siemens Concentriq Dx detection system (Siemens +/HRP/Mo, Germany). We determined the immunostaining degree according to the proportion of positively stained tumor cells (0: 0-5%; 1: 6-25%; 2: 26-50%; 3: 51-75% and 4: 76-100%) and the intensity of staining (0: no staining; 1: weak staining; 2: moderate staining and 3: strong staining). We calculated the final expression score by combining both scores as previously described 10.

### 2.11. Immunofluorescence staining

Sections of tumor tissue from GC patients and nude mouse subcutaneous xenograft models were fixed with 4% paraformaldehyde and blocked for 30 minutes in 20% goat serum, then rehydrated in PBS, and overnight incubation with primary antibodies is followed. Then, a humidified chamber was used to incubate the slides for 1 hour with fluorescent secondary antibodies after being washed three times in PBS. Subsequently, the nuclei of the cells were counterstained with 4′, 6-diamidino-2-phenylindole (DAPI). The images were taken with a Zeiss LSM 510 META confocal fluorescence microscope.

### 2.12. Single cells isolated from GC tissues

Hank's solution was used to wash freshly cut subcutaneous xenograft GC tissues three times before they were cut into small pieces. Collagenase IV and DNase I were added to RPMI-1640 medium and the specimens were further incubated at 37°C under continuous rotation for 1-2 h. Following three washes with PBS, the cell suspensions were filtered with a 70 mm cell strainer (BD Labware). Typically, trypan blue exclusion staining revealed a cell viability of more than 90%.

### 2.13. Isolation of neutrophils from peripheral blood

Based on the manufacturer's instructions, we isolated neutrophils from peripheral blood of healthy donors with the Human Neutrophil Isolation Kit (LZS11131). The isolated cells that were used had a viability of >90% and a purity of >95%.

### 2.14. Flow cytometry

Single GC tumor cells were prepared by the procedure mentioned above and then incubated on ice for 30 minutes with Fc block (anti-mouse CD16/32, Abcam). Supplementary Table 3 shows the details regarding the corresponding flow cytometry antibodies that were added according to the type of immune cells to be detected.

The cell cycle and apoptosis assays were performed using Cell Cycle Staining Kit (70-CCS012, MultiSciences), and Annexin V-FITC/PI Apoptosis Kit (70-AT101-100, MultiSciences), respectively.

Single-cell suspensions from tumor tissues were stained with antibodies against CD45, CD49b, CD11b, F480, CD206, CD86, Ly6C and Ly6G (all from BioLegend) after Fc receptor blocking. Data were acquired on a BD LSRFortessa and analyzed with FlowJo v10.8. The gating strategy included sequential selection of cells (FSC/SSC), singlets (FSC-A/FSC-H), live cells (SSC-A/ Comp-APC-Cy7-A), and CD45⁺ leukocytes. Immune subsets were identified as: macrophages (CD45⁺F480⁺), MDSCs (SSC-A/CD11b^+^), NK (CD49b⁺CD11b⁺) and neutrophils (CD45⁺CD11b⁺). FMO controls were used to set positivity gates. Data are presented as percentages of the parent population.

### 2.15. Conditioned medium (CM) preparation

Conditioned medium of cancer cells (MKN45 and NCI-N87 with overexpression or underexpression NOX4) was prepared according to the protocol of a previous study 16. In brief, cancer cells were seeded in 6-well plates and grown for 48 hours before being harvested as supernatants for neutrophil treatment. Neutrophil activation was induced by replacing the conditioned medium after 24 hours.

### 2.16. Survival assay for neutrophils

Healthy donor neutrophils were conditioned by incubating with the conditioned medium of MKN45 and NCI-N87 with overexpression or underexpression NOX4 for 16 hours. According to the manufacturer's instructions, survival of neutrophils was assessed using Annexin V-FITC/PI Apoptosis Kit.

### 2.17. *In vitro* neutrophil migration and activation assays

The migration of neutrophils was assessed using FalconTM Cell Culture Inserts that contain polycarbonate membranes with 1-micron pore sizes. A 24-well plate was seeded with neutrophils (1×10^5^ cells/well) in the upper chamber of a transwell. Lower chambers were filled with conditioned medium from MKN45, MKN45-NOX4-OE, MKN45-NOX4-KD, NCI-N87 or NCI-N87-NOX4-KD cells. After 16 hours, a cotton swab was carefully used to clean the upper surface of the filter after the cell suspension in the upper chamber had been removed. Neutrophils were fixed and stained with crystal violet, and images from five representative fields of each membrane were captured.

Activation of neutrophils was evaluated according to changes in cell morphology, prolongation of cell lifespan, enhancement of cell viability, decreases in cell apoptosis and increases in the expression of PD-L1 and arginase 1 (Arg-1).

### 2.18. Cytokine antibody array

Human XL Cytokine Array (ARY022B, R&D) was used to measure the levels of cytokines secreted by neutrophils after treatment with conditioned medium from MKN45 cells with overexpression or underexpression NOX4. To identify the cytokines with significant differences in expression, gray values were calculated semiquantitatively using Image-Pro software.

### 2.19. ELISA

Conditioned medium from MKN45, MKN45-NOX4-OE, MKN45-NOX4-KD, NCI-N87 and NCI-N87-NOX4-KD cells cocultured with peripheral blood neutrophils from healthy donors and plasma specimens from 30 patients with newly diagnosed GC before surgery or treatment were collected to test the expression of NOX4, CD14, GM-CSF, VEGF, IL-15 and DKK-1 by ELISA. All assays were performed using commercial ELISA kits (NOX4: GWB-KBBWB4, CD14: GWB-KBBWB9, GM-CSF: bsk12028, VEGF: bsk13007, IL-15: GWB-KBBWB8 and DKK-1: GWB-KBBWB6) according to the manufacturer's instructions.

### 2.20. Neutrophil adhesion assay

Neutrophils from healthy donors treated with conditioned medium from MKN45 and NCI-N87 cells with overexpression or underexpression NOX4 for 16 h were diluted with calcein-AM (C2012-0.1 mL, Beyotime) working solution to 2~5×10^6^ cells/mL and incubated for 30 min in dark at 37°C. Then, the samples were centrifuged at 500 × g for 5 min, the supernatant was discarded, and the neutrophils were washed twice with PBS prewarmed to 37°C. Subsequently, the cells were resuspended in phenol red-free RPMI 1640 prewarmed to 37°C with density :2~5x10^6^ cells/mL, and 500 μL of the sample was added to a 6-well plate of HUVECs with a confluency of approximately 80%; An incubator with 5% CO2 and 37°C was used to incubate the cells for one hour. Finally, we gently washed the 6-well plates twice with PBS, and pictured of 5 random fields of view captured under a fluorescence microscope.

### 2.21. RNA sequencing (RNA-seq) analysis of neutrophils

Total RNA was extracted from neutrophils pretreated with conditioned medium of MKN45, MKN45-NOX4-OE, MKN45-NOX4-KD, NCI-N87 and NCI-N87-NOX4-KD GC cells using TRIzol (Invitrogen). Then, total RNA was subjected to library preparation, and the data was analyzed. Significant differential expression of a gene was defined as a > 2-fold expression difference vs the control with an adjusted P value less than 0.05. A heat map was analyzed by Gene Ontology (GO) using Cluster software and visualized with Java Tree View. Differentially expressed genes (DEGs) were analyzed by GO using the AMIGO and DAVID software. The enrichment degrees of DEGs were analyzed using Kyoto Encyclopedia of Genes and Genomes annotations (KEGG). The raw RNA-seq data had been deposited to the Genome Sequence Archive-Human (GSA- Human) database. The data is accessible with the accession number [HRA014121]. [https://ngdc.cncb.ac.cn/asahuman/browse/HRA014121].

### 2.22. High-resolution non-target metabolic mass spectrometry

Neutrophils freshly extracted from the peripheral blood of healthy donors were cocultured with conditioned medium of MKN45, MKN45-NOX4-OE, MKN45-NOX4-KD, NCI-N87 and NCI-N87-NOX4-KD GC cells for 24 h. A 2:2:1 methanol, acetonitrile, and ddH_2_O_2_ solution was added to the oscillator and mixed. The samples were cryogenically sonicated at 4°C for 30 min, incubated at -20°C for 10 min, and centrifuged at 14000 × g for 20 min at 4°C. Under vacuum, the supernatant was dried. Then, 100 μL of acetonitrile/ddH_2_O_2_ solution (1:1) was added to the sample for redissolution, the sample was centrifuged at 14000 × g for 15 min at 4°C, and analyses were conducted on the supernatant. An Agilent 1290 Infinity LC ultra-high-pressure liquid chromatography (UHPLC) system HILIC column was used to separate the samples. After that, a Triple TOF 6600 mass spectrometer (AB SCIEX) was used to perform mass spectrometry. After XCMS data extraction, preprocessing, quality control evaluation, and analysis, metabolite structure was identified.

### 2.23. Statistical analysis

Mean ± SD is used to represent all experimental data. The Student's t test was used to compare the differences between two sets. The multiple comparisons were performed using a one-way analysis of variance (ANOVA). A chi-square test was performed for analysis of variance. An analysis of linear regression was conducted to determine the correlation between two variables. An analysis of the overall survival curves was performed using the Kaplan-Meier method and the log-rank test was used to compare them. A Cox proportional hazard regression model was used in a stepwise manner for both univariate and multivariate analyses, and Statistical significance was considered at p0.05 (*p < 0.05, **p < 0.01). SPSS 24.0 software was used to perform the statistical analyses.

## 3. Results

### 3.1. High expression of NOX4 correlated with poor clinical outcome

**Figure 1:**
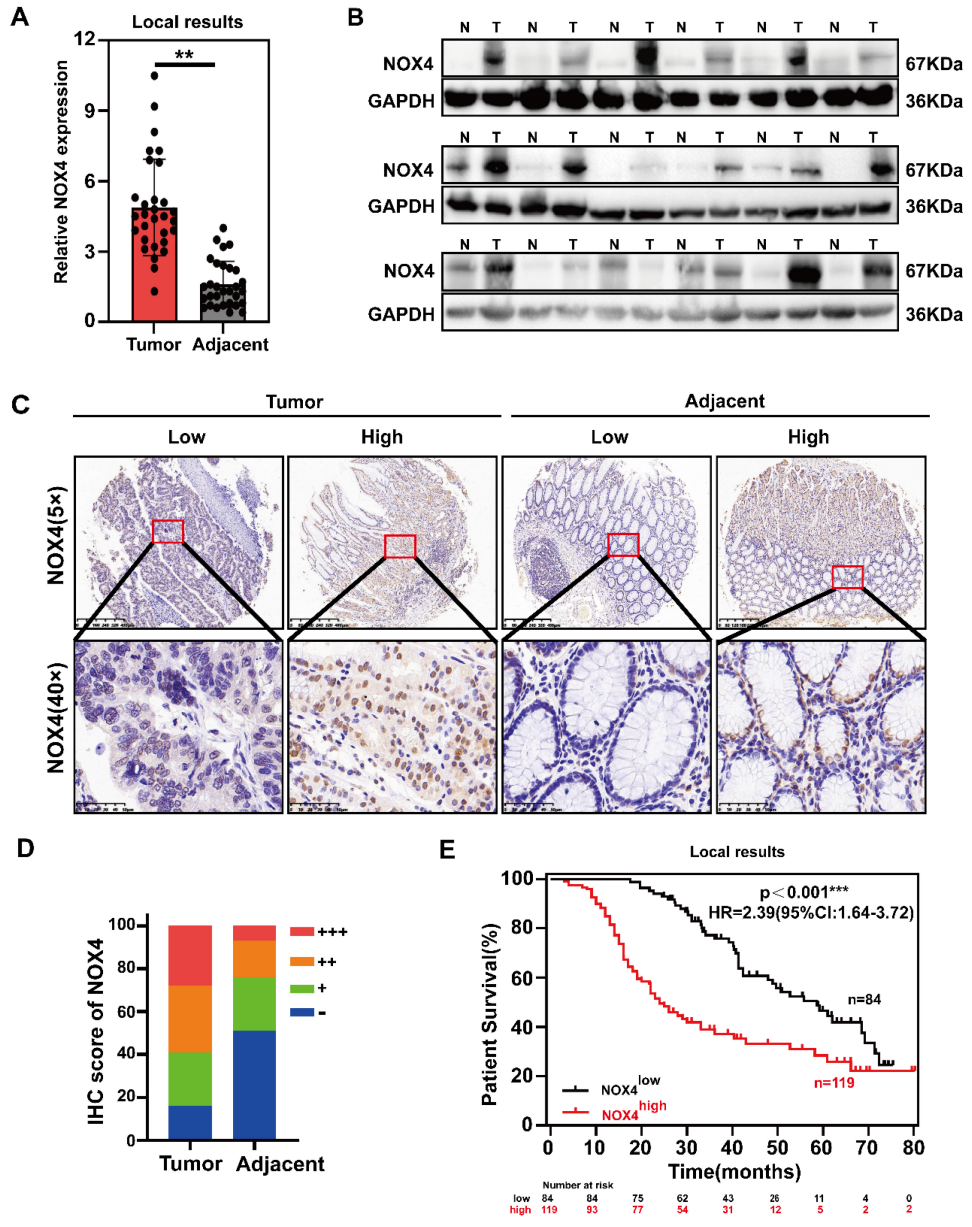
** NOX4 is overexpressed in GC and correlated with poor prognosis.** (A) mRNA expressions of NOX4 in tumor and adjacent tissues were detected by RT-PCR from 30 paired GC of Ruijin Hospital. (B) Western blot analysis of NOX4 expression in GC tissues compared with corresponding adjacent gastric tissues. N, normal tissue; T, tumor tissue. (C, D) Representative images and statistical analysis of NOX4 IHC staining intensity in 203 paired tissues. (E) Kaplan-Meier overall survival curves for GC tissues with high and low NOX4 levels in Ruijin cohort (n=203). The data represent three independent experiments. Data are presented as the mean ± SD. *P < 0.05; **P < 0.01; ***P< 0.001.

To determine NOX4 expression in GC, we first used the GEPIA database and 30 pairs of human GC and adjacent specimens to analyze the differences in mRNA levels. According to the results, GC had significantly higher NOX4 expression than adjacent gastric tissue (Figure 1A and Supplementary Figure 1A). Wb and IHC analysis of GC patient samples confirmed the up-regulation of NOX4 protein expression (Figure 1B-1D). Additional data retrieved from the GEPIA database demonstrated that NOX4 expression was enhanced in other solid cancers, including diffuse large B-cell lymphoma (DLBC), glioblastoma (GBM), head and neck squamous cell carcinoma (HNSC) and pancreatic adenocarcinoma (PDAC) (Supplementary Figure 1A). As well, GC cell lines expressed higher levels of NOX4 protein and mRNA than GES-1 normal gastric cells (Supplementary Figure 1B-1C).

IHC was used to examine the expression of NOX4 in 203 GC tissues to further investigate its clinical significance. All patients were divided into NOX4^high^ (++ and +++) and NOX4^low^ (- and +) groups. The typical immunostaining images of NOX4 in tumor and adjacent tissues were presented in Figure 1C, showing minimal intensity in nontumor gastric and obvious up-regulation in tumor tissues (Figure 1D). There was a poorer prognosis for patients with high NOX4 expression (Figure 1E). Further analysis of the correlation between NOX4 expression and clinicopathological features revealed that high NOX4 expression was positively correlated with TNM stage (p=0.023), N stage (p=0.015), tumor size (p=0.042), vascular invasion (p=0.003), and pathological differentiation grade (p=0.001) (Supplementary Table 4). Moreover, a Cox proportional hazard model indicated that NOX4 expression was a significant independent predictor of GC patient survival. Additionally, tumor size and TNM stage also determined prognosis (Supplementary Figure 1E). In light of the above evidence, we concluded that compared to nontumor, NOX4 expression in GC is high and may signify a dismal clinical outcome and tumor aggressiveness.

### 3.2. Tumor-derived NOX4 promoted intratumoral infiltration of neutrophils

**Figure 2:**
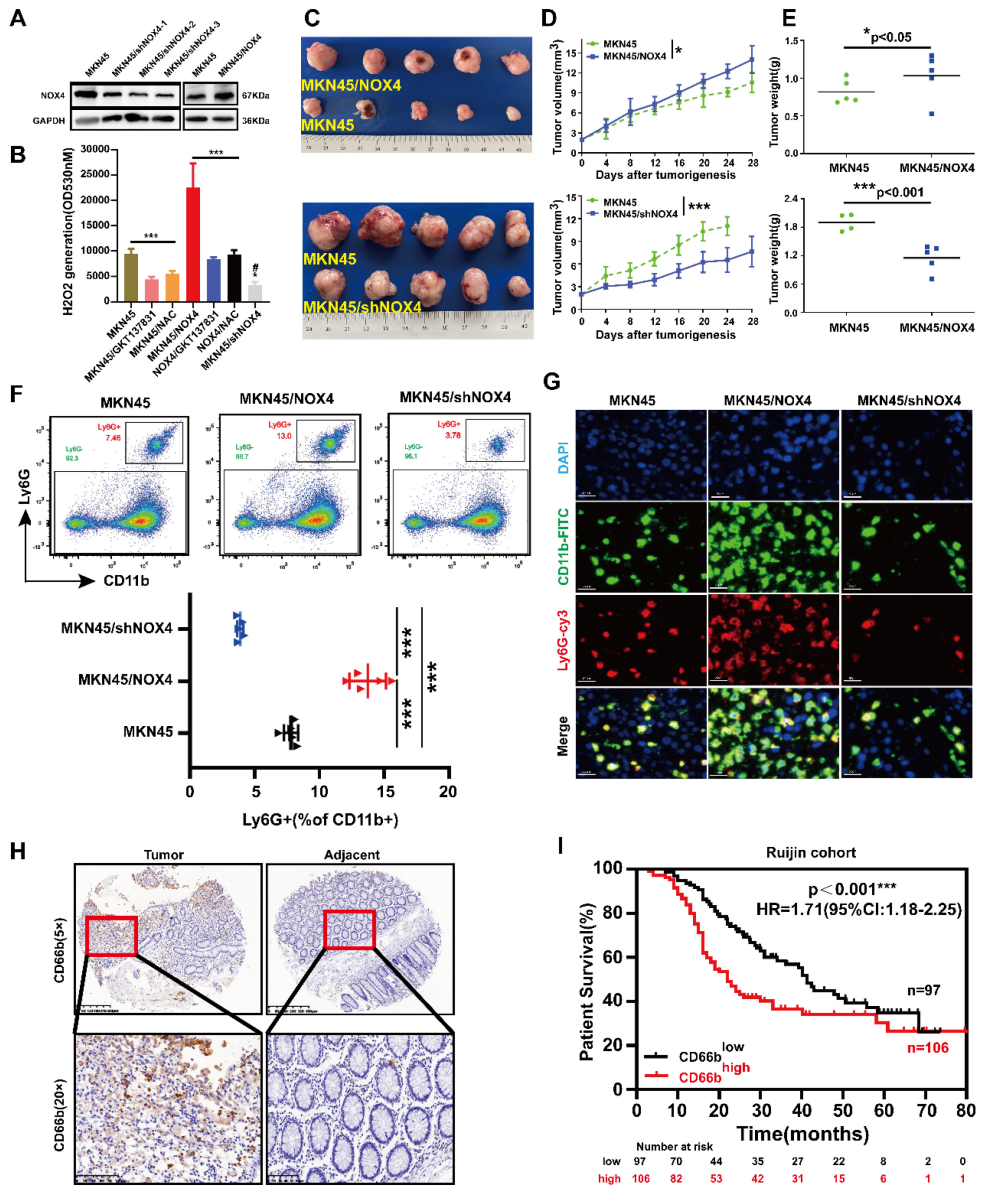
** Tumor-derived NOX4 promotes intratumoral infiltration of neutrophils.** (A) Western blot analysis of NOX4 overexpression and knockdown in MKN45 GC cell line. (B) Production of H2O2 of MKN45, MKN45-NOX4-OE and MKN45-NOX4-KD cells treated with GKT137831 (20μM) or NAC (2.5mM). (C-E) Tumor growth curves and tumor burdens in immunodeficient nude mice injected subcutaneously with MKN45, MKN45-NOX4-OE and MKN45-NOX4-KD cells (n=5). Data points are shown only up to the time point at which the average tumor volume in any group first reached the predefined ethical limit. Mice were euthanized thereafter. (F) Quantification of tumor-infiltrating neutrophils analyzed by flow cytometry using the surface markers of CD11b and Ly6G on MKN45, MKN45-NOX4-OE and MKN45-NOX4-KD cells tumors grafted into BALB/C nude mice. (G) Quantification of tumor-infiltrating neutrophils analyzed by Immunofluorescence staining using the surface markers of CD11b and Ly6G on MKN45, MKN45-NOX4-OE and MKN45-NOX4-KD cells tumors grafted into BALB/c nude mice. (H) Representative images of CD66b IHC staining intensity in 203 paired tissues. (I) Kaplan-Meier overall survival curves for GC tissues with high and low CD66b levels in 203 GC patients. The data represent three independent experiments. Data are presented as the mean ± SD. *P < 0.05; **P < 0.01; ***P< 0.001; # compared with MKN45, P < 0.05.

In MKN45, a line with medium expression of NOX4, NOX4 was forced to overexpress by plasmids and down-regulated by lentiviruses. NCI-N87, a line with high expression of NOX4, NOX4 was down-regulated. NOX4 could be significantly knocked down by shNOX4-3 in MKN45 cells and by shNOX4-2 in NCI-N87 cells (Figure 2A and Supplementary Figure 2A). The interference efficiency of NOX4 was verified by ROS assays (Figure 2B and Supplementary Figure 2B). NOX4 promoted the growth of subcutaneous xenografts in BALB-c nude mice (Figure 2C-2E and Supplementary Figure 2C-2E). To explore the correlation between NOX4 and immune cells in tumors, we profiled immune cells in tumors by flow cytometry (Supplementary Figure 3). We observed that the number of infiltrating neutrophils in tumors were significantly correlated with the expression of NOX4 (MKN45 vs MKN45/NOX4 vs MKN45/shNOX4: 7.67% ± 1.8% vs. 13.49% ± 2.9% vs. 3.79% ± 1.5%, P<0.005***. Figure 2F-2G and Supplementary Figure 2F-2G). To provide a more comprehensive view, we extended our analysis to a panel of additional established neutrophil markers (CD177, FCAR and CSF3R), all of which showed significant positive correlation with NOX4 (Supplementary Figure 2H), further reinforcing this association.

To determine whether the correlation between NOX4 expression and tumor-infiltrating neutrophils in the xenograft model was also present in human GC samples, IHC to detect CD66b was performed on the 203 GC samples mentioned above (Supplementary Figure 2I). Furthermore, we assessed the expression of *CEACAM8*, which encodes the neutrophil-specific marker CD66b, in 30 pairs of gastric cancer tissues. As shown in Figure 2H and Supplementary Figure 1D, the number of neutrophils infiltrating the tumor was significantly higher than that in the adjacent tissue. Variable levels of *CEACAM8* transcript were detected across the samples, corroborating the infiltration of neutrophils in these tumors at the transcriptional level. The patients were divided into CD66b^high^ infiltration group and CD66b^low^ infiltration group according to the average number of cells/high-power field (HPF) (21 cells). The correlations between CD66b^+^ cell infiltration and clinicopathological features of patients are shown in Supplementary Table 5. CD66b^+^ cell infiltration was associated with TNM stage (P=0.024), M stage (P=0.030), tumor size (P=0.034) and NOX4 expression (P=0.025). Furthermore, patients in the CD66b^high^ infiltration group had a significantly poorer prognosis than those in the CD66b^low^ infiltration (Figure 2I). Moreover, univariate and multivariate Cox proportional hazard analyses suggested that high neutrophil infiltration was an independent predictive indicator of the prognosis of GC patients (Supplementary Figure 1E). Taken together, these results suggested that tumor derived NOX4 recruited neutrophil infiltration within GC, but the underlying mechanism is unclear.

### 3.3. Tumor-derived NOX4 induced neutrophil activation into a tumor-promoting phenotype

**Figure 3:**
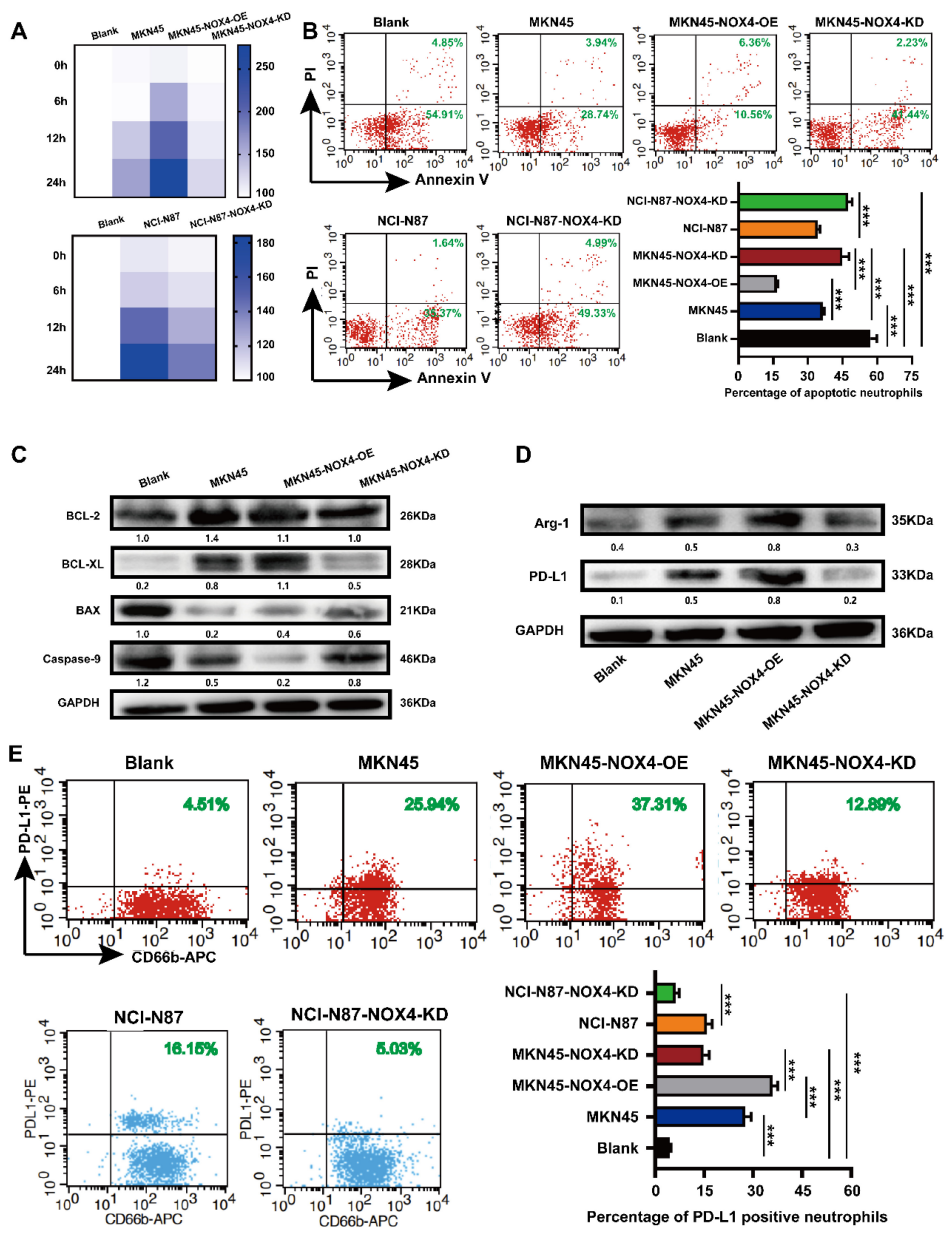
** Tumor-derived NOX4 sustains neutrophils survival, activation, PD -L1 and Arg-1 expression.** (A) Heat map showed the viability of neutrophils conditioned by conditioned medium of MKN45, MKN45-NOX4-OE, MKN45-NOX4-KD, NCI-N87 and NCI-N87-NOX4-KD GC cells for 0h, 6h, 12h and 24h. (B) Dot plots and statistics analysis of neutrophil apoptosis was conditioned by conditioned medium of MKN45, MKN45-NOX4-OE, MKN45-NOX4-KD, NCI-N87 and NCI-N87-NOX4-KD GC cells for 16h. (C) Western blot analysis of apoptosis-related protein expression in neutrophils conditioned by conditioned medium of MKN45, MKN45-NOX4-OE and MKN45-NOX4-KD cells. (D) Western blot analysis of PD-L1 and Arg-1 protein expression in neutrophils conditioned by conditioned medium of MKN45, MKN45-NOX4-OE and MKN45-NOX4-KD cells. (E) Dot plots analysis of PD-L1 expression in neutrophils conditioned by conditioned medium of MKN45, MKN45-NOX4-OE, MKN45-NOX4-KD, NCI-N87 and NCI-N87-NOX4-KD GC cells. The data represent three independent experiments. Data are presented as the mean ± SD. *P < 0.05; **P < 0.01; ***P< 0.001.

To better explore the correlation between tumor-derived NOX4 and neutrophils, we first purified neutrophils with a commercial human peripheral blood neutrophil extraction kit and identified them by flow cytometry (Supplementary Figure 4A-4B). Purified peripheral blood neutrophils from healthy donors showed obvious morphological changes after stimulation by GC cell- conditioned medium, such as uneven size, swollen and spindle-shaped cell bodies, pseudopodia and contact-independent growth (Supplementary Figure 4C). According to the literature 15, neutrophils with the above characteristics were considered to be in an activated state. Neutrophils conditioned by conditioned medium from GC cells with NOX4 overexpression showed significantly prolonged lifespan (Figure 3A), decreased apoptosis (Figure 3B-3C), and up-regulated expression of PD-L1 and Arg-1 (Figure 3D-3E). Conversely, when neutrophils were conditioned with conditioned medium from NOX4-KD GC cells, their lifespan were shortened, apoptosis were increased, and expression of PD-L1 and Arg-1 were decreased compared with those of the control group. These findings together implied that tumor-derived NOX4 was involved in the activation of neutrophils as well as PD-L1 expression.

Further functional verification of the activated neutrophils showed that their own chemotaxis and adhesion to HUVECs were significantly enhanced (Supplementary Figure 5A-5D). Furthermore, we also found that the activated neutrophils significantly inhibited MKN45 and NCI-N87 GC cell apoptosis, especially in the NOX4 overexpression group (Supplementary Figure 5E). Interestingly, studies to determine the effects of NOX4 on biological functions of GC cells showed that activated neutrophils could significantly enhance the migration of GC cells but had no obviously effect on their cell cycle or clone formation (Supplementary Figure 6). To substantiate these pro-tumor effects *in vivo*, we established a pulmonary metastasis model by co-injecting GC cells with activated or control neutrophils into NOD/SCID mice. Consistent with the *in vitro* findings, NOX4-activated neutrophils significantly increased the number of metastatic nodules in the lungs compared to controls, an effect that was reversed by the NOX1/4 inhibitor GKT137831 (Supplementary Figure 7A and Supplementary Figure 7D). Immunohistochemical analysis of the metastatic lesions further revealed increased intratumoral neutrophil infiltration (CD66b^+^) and enhanced tumor cell proliferation (Ki67^+^) in the NOX4-activated neutrophil group (Supplementary Figure 7B-7C and Supplementary Figure 7E-7F). These results suggested that neutrophils activated by tumor-derived NOX4 had pro-tumor function.

### 3.4. Tumor-derived NOX4 recruited neutrophils to the tumor microenvironment via GM-CSF

**Figure 4:**
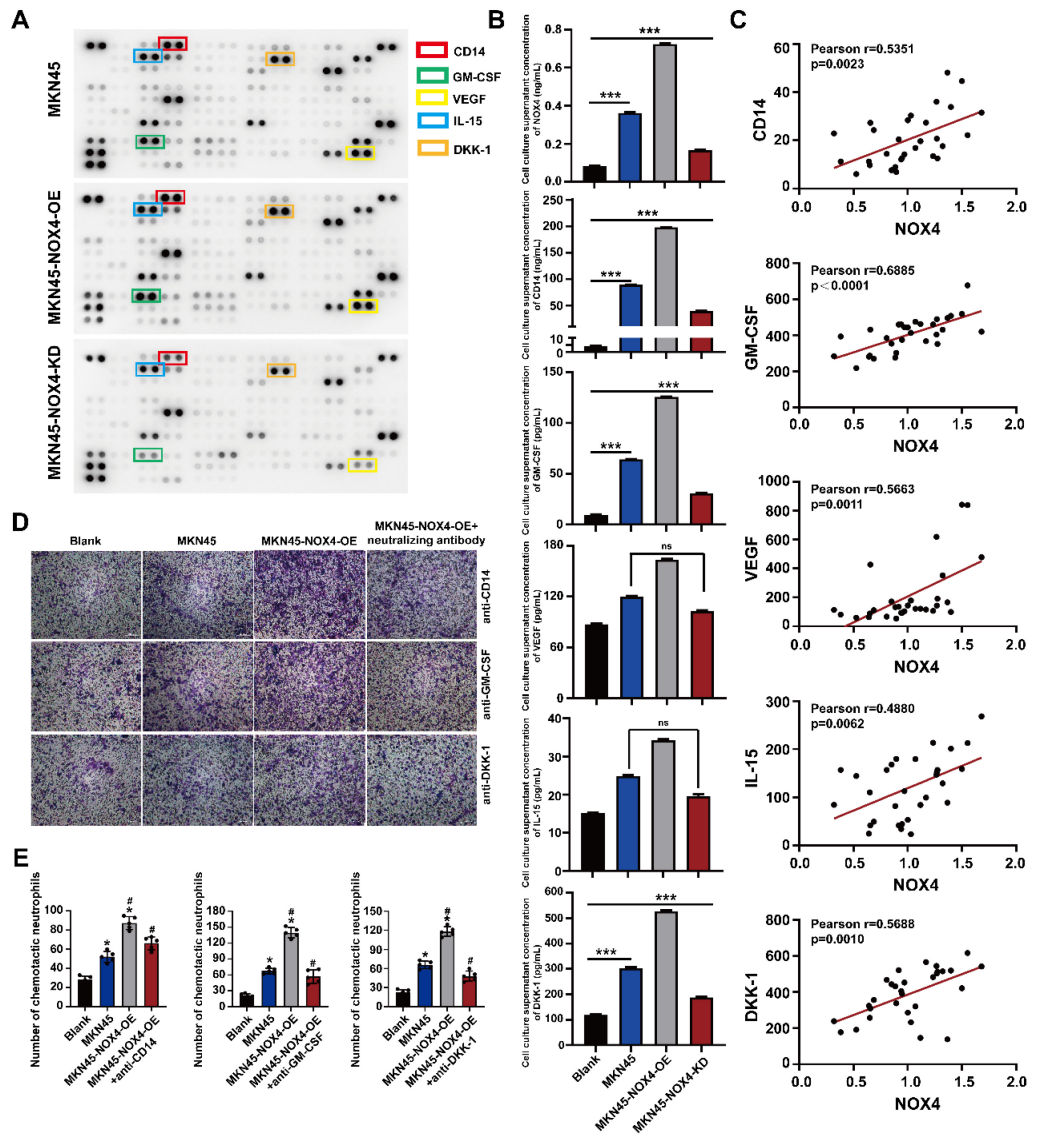
** Various cytokines are involved in the recruitment of neutrophils.** (A) Cytokine array of the conditioned medium of healthy donor neutrophils and MKN45, MKN45-NOX4-OE and MKN45-NOX4-KD cells. (B) NOX4, CD14, GM-CSF, VEGF, IL-15 and DKK-1 were verified by ELISA in the co-culture supernatants of MKN45, MKN45-NOX4-OE and MKN45-NOX4-KD cells and healthy donor neutrophils. (C) NOX4, CD14, GM-CSF, VEGF, IL-15 and DKK-1 were verified by ELISA in serum of 30 patients with newly diagnosed GC. (D, E) Added the anti-CD14 (20μg/ml), anti-GM-CSF (20μg/ml) or anti-DKK-1 (20μg/ml) into the conditioned medium of MKN45-NOX4-OE GC cell, neutrophils chemotactic capacity were evaluated by the transwell assay. The data represent three independent experiments. Data are presented as the mean ± SD. *P < 0.05; **P < 0.01; ***P< 0.001.

To reveal the key molecules involved in NOX4 chemotactic neutrophils infiltration, we sought to determine the effect of NOX4 on chemokine expression in GC cells through cytokine screening by using a Human XL cytokine array. As shown in Figure 4A, several cytokines and chemokines showed different levels in the culture medium of neutrophils co-cultured with MKN45, MKN45-NOX4-OE and MKN45-NOX4-KD cells, and among these factors, CD14, GM-CSF, VEGF, IL-15 and DKK-1 were the most significantly different. Next, the levels of the above five factors in the co-culture supernatants of MKN45, MKN45-NOX4-OE, MKN45-NOX4-KD, NCI-N87 and NCI-N87-NOX4-KD cells and neutrophils and the serum of 30 patients with newly diagnosed GC were further verified by ELISA. As shown in Figure 4B-4C and Supplementary Figure 8A, CD14, GM-CSF and DKK-1 concentration were positively correlated with NOX4 expression. Furthermore, added anti-CD14 (20μg/ml), anti-GM-CSF (20μg/ml) or anti-DKK-1 (20μg/ml) antibodies to the supernatant of MKN45-NOX4-OE cells partially abrogated the enhancement of neutrophil migration induced by NOX4 (Figure 4D-4E and Supplementary Figure 8B). Moreover, the concentration of NOX4 in the plasma of 30 GC patients detected by ELISA was positively correlated with the number of neutrophils in peripheral blood. These results indicated that CD14, GM-CSF and DKK-1 may be involved in recruiting neutrophils.

**Figure 5:**
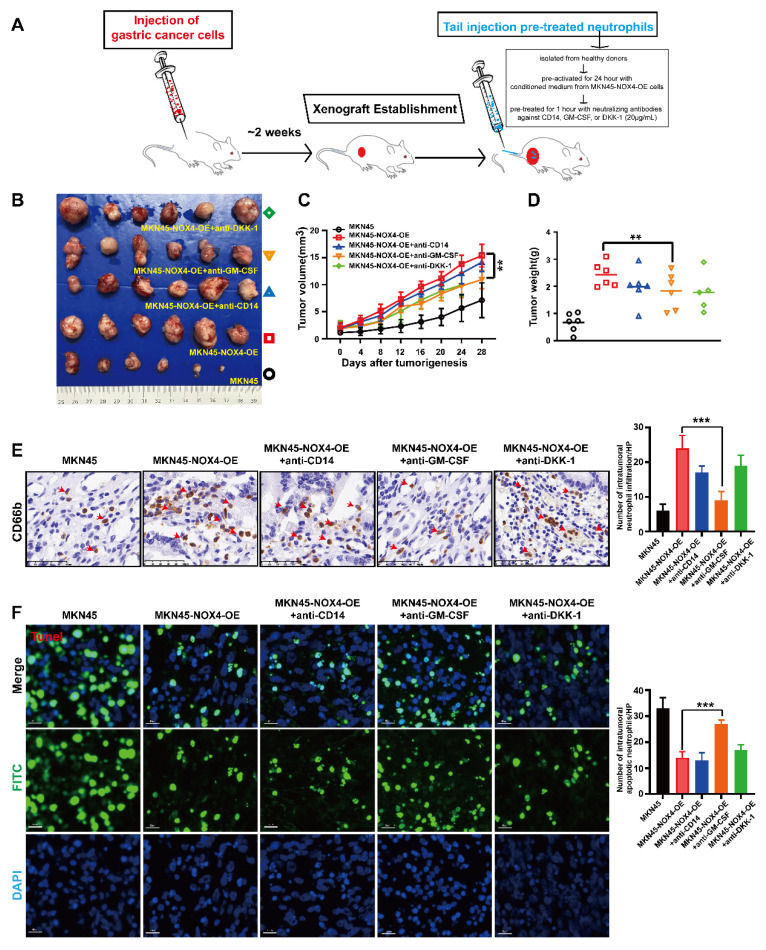
***In vivo* experiments confirmed that tumor-derived NOX4 inhibit the apoptosis of GC cells by recruiting neutrophils into the tumor microenvironment through GM-CSF.** (A) Tumor formation and treatment model of NOD/SCID mice. (B-C) NOD/SCID mice (6 per group) were inoculated subcutaneously with NOX4-overexpression MKN45 GC cell. Once tumor size reached 2mm^3^, 5×10^6^/200μL of neutrophils from healthy donors and pre-treated with different chemokine neutralizing antibodies (anti-CD14, anti-GM-CSF and anti-DKK1, respectively) were injected into the tail vein. Tumor growth curves (C) and tumor burdens (D) plotted from data measured every 4 days. Data points are shown only up to the time point at which the average tumor volume in any group first reached the predefined ethical limit. Mice were euthanized thereafter. (E) Neutrophils infiltration in tumors determined by CD66b IHC in each group. The red arrow refers to neutrophils. (F) Apoptosis of gastric cancer cells determined by Tunel Apoptosis Detection Kit in each group. Data are presented as the mean ± SD. *P < 0.05; **P < 0.01; ***P< 0.001.

To validate the functional impact of NOX4 on neutrophil infiltration and tumor progression *in vivo*, we established subcutaneous xenograft models using control and NOX4-overexpressing MKN45 cells. As illustrated in Figure 5A-5D, NOX4 overexpression significantly promoted tumor growth compared to the vector control. Immunohistochemical analysis further revealed a marked increase in neutrophil infiltration in NOX4-overexpressing tumors (24 ± 3.7 vs. 6 ± 1.9 per HPF, *p* < 0.001), which was accompanied by a pronounced reduction in tumor cell apoptosis (15 ± 2.2 vs. 34 ± 3.2 TUNEL-positive cells per HPF, *p* < 0.001; Figure 5E-5F).

To dissect the contribution of specific chemokines to neutrophil recruitment, we performed *in vivo* neutralization experiments (Figure 5A). Neutrophils were pre-treated with neutralizing antibodies against CD14, GM-CSF, or DKK-1 prior to tail vein injection into tumor-bearing mice. While pre-treatment with anti-CD14 and anti-DKK-1 led to a modest, non-significant reduction in neutrophil infiltration (17 ± 1.9 and 19 ± 3.0 cells/HPF, respectively; Figure 5E), only GM-CSF neutralization resulted in a statistically significant inhibition of neutrophil recruitment (9 ± 2.3 vs. 24 ± 3.7 cells/HPF in the isotype control, *p* < 0.001). Consistently, only the GM-CSF neutralization group showed a significant increase in tumor cell apoptosis (33 ± 4.1 vs. 15 ± 2.2 TUNEL-positive cells/HPF, *p* < 0.001; Figure 5F), to a level comparable to that observed in the vector control group. Collectively, these *in vivo* findings demonstrate that GM-CSF is the dominant factor responsible for NOX4-dependent neutrophil recruitment and the subsequent suppression of apoptosis in the tumor microenvironment.

### 3.5. Neutrophils chemotactically recruited into the tumor microenvironment by tumor-derived NOX4 underwent significant metabolic remodeling

**Figure 6:**
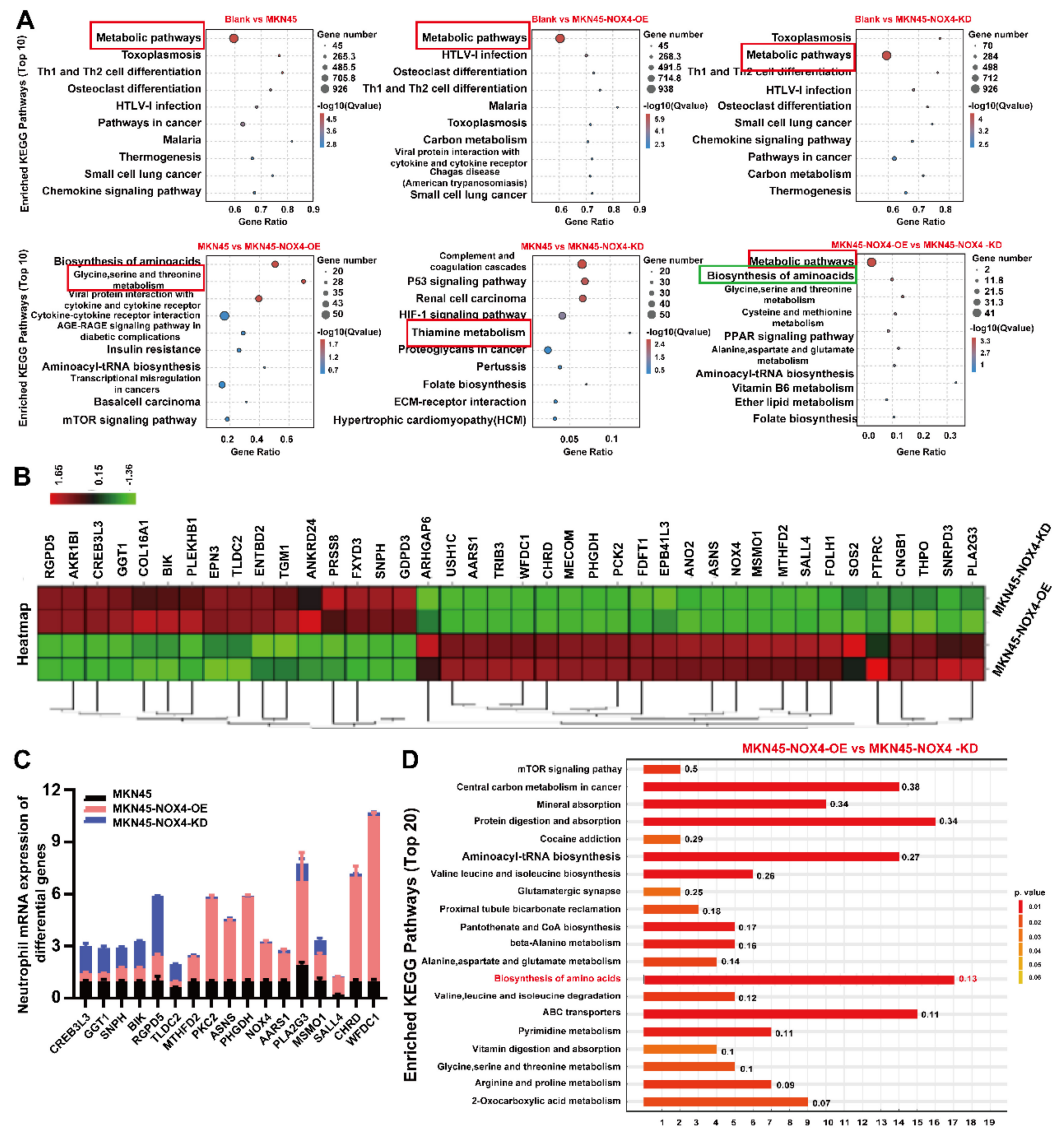
** Tumor-derived NOX4 conditioned neutrophils underwent metabolic remodeling.** (A) KEGG pathway analysis of RNA-sequencing showed the significantly differences in metabolic signaling pathways of neutrophils in each group. (B) The 40 genes with the most significant differences in neutrophils conditioned by conditioned medium of MKN45-NOX4-OE and MKN45-NOX4-KD cells. (C) qRT-PCR was used to verify the above 40 differential genes in neutrophils conditioned by conditioned medium of MKN45, MKN45-NOX4-OE and MKN45-NOX4-KD cells. The data represent three independent experiments. Data are presented as the mean ± SD. *P < 0.05; **P < 0.01; ***P< 0.001. (D) KEGG pathway analysis of non-targeted metabolic mass spectrometry. Y-axis: Represents the specific metabolic pathways annotated from the KEGG database. X-axis: Represents the Rich Factor. Numbers on the bars: Display the p-value for each pathway.

To clarify the mechanism of neutrophil chemotaxis to the tumor microenvironment promotes tumorigenesis, we detected the levels of transcription in neutrophils treated with conditioned medium from MKN45, MKN45-NOX4-OE, MKN45-NOX4-KD, NCI-N87 and NCI-N87-NOX4-KD cells. As shown in Figure 6A and Supplementary Figure 9A, significant changes in metabolic pathways were observed between groups. To further identify the effective genes, qRT‒PCR were used to validate the top 40 genes between the MKN45 NOX4-OE and MKN45 NOX4-KD groups (Figure 6B). Among the 17 validated genes, 11 (MTHFD2, PKC2, ASNS, PHGDH, NOX4, AARS1, PLA2G3, MSMO1, SALL4, CHRD and WFDC1) increased in line with NOX4 expression, and six (CREB3L3, GGT1, SNPH, BIK, RGPD5 and TLDC2) did the opposite (Figure 6C). Next, in order to validate the above results from a multi omics perspective, we conducted a non-targeted metabolic mass spectrometry evaluation of neutrophils treated with conditioned medium from MKN45, MKN45-NOX4-OE, MKN45-NOX4-KD, NCI-N87 and NCI-N87-NOX4-KD, cells. The results showed significant differences in neutrophil metabolism between groups (Supplementary Figure 9B). KEGG pathway enrichment hinted that the biosynthesis of amino acids pathway was significantly activated (Figure 6D and Supplementary Figure 9C).

**Figure 7:**
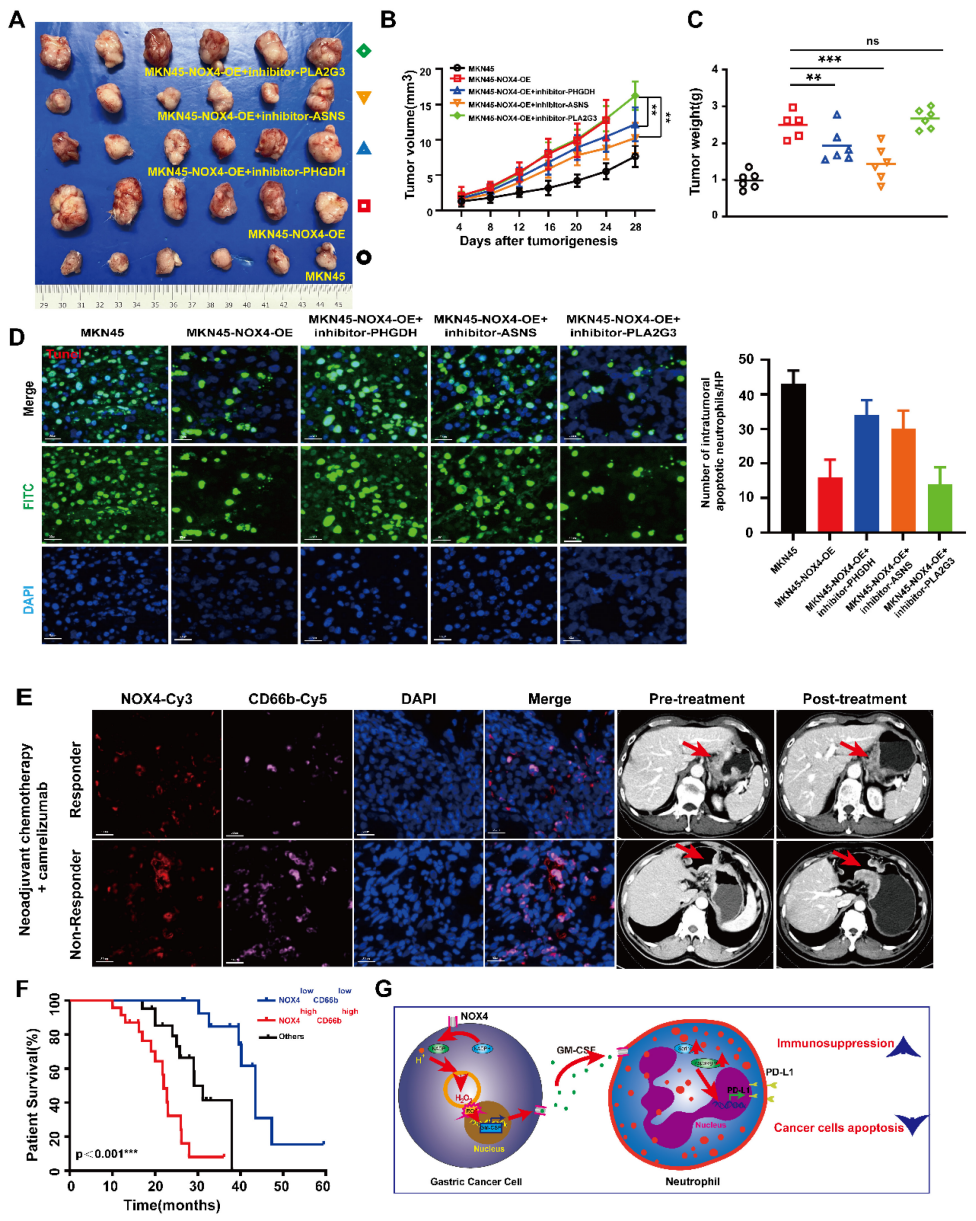
** Targeting neutrophil amino acid metabolism and tumor NOX4 can inhibit tumor growth* in vivo*.** (A-C) NOD/SCID mice (6 per group) were inoculated subcutaneously with NOX4-overexpression MKN45 GC cell. Once tumor size reached 2mm^3^, 5×10^6^/200μL of neutrophils from healthy donors and pre-treated with inhibitor of PHGDH (used at 15μM), inhibitor of ASNS (used at 20μM) and inhibitor of PLA2G3 (used at 50μM) were injected into the tail vein. Tumor growth curves (B) plotted from data measured every 4 days. (C) Tumor burdens plotted. (D) Apoptosis of gastric cancer cells determined by Tunel Apoptosis Detection Kit in each group. Data are presented as the mean ± SD. *P < 0.05; **P < 0.01; ***P< 0.001. (E) Immunofluorescence staining analysis of NOX4 expression and neutrophils infiltration in pre-treatment tumors are shown. Locations of primary tumors were identified by Computed Tomography in GC patients treated with anti-PD-1 therapy (carelizumab). (F) Kaplan-Meier curves show significantly improved overall survival in patients positive for the signature within the immunotherapy cohort (n=59, log-rank test). (G)Schematic representation of the proposed working model. NOX4 inhibition alleviates the immune suppression tumor microenvironment and elevates the apoptosis of gastric cancer cell by recruiting and modifying neutrophils.

Given the above results showed that the biosynthesis of amino acid pathway was the most obvious difference between groups, and non-target metabolomics also found that this metabolic pathway is obviously activated, combined with the results of qRT-PCR, we focused on genes related to amino acid metabolism (PHGDH, ASNS and PLA2G3) for subsequent functional validation. As shown in Supplementary Figure 10A-10B, the viability of neutrophils treated with conditioned medium from MKN45-NOX4-OE cells combined with inhibitors of the above three genes were decreased to varying degrees, accompanied by increased apoptosis, and there were significant difference in the anti-PHGDH and anti-ASNS groups. In addition, MKN45-NOX4-OE cells were subcutaneously inoculated into NOD/SCID mice. After tumor formation (approximately one week), neutrophils pretreated with inhibitors of the above three genes were injected into the tail vein at a dose of 5×10^6^ cells/200 μL. The results showed that the inhibition of amino acid synthesis in neutrophils could inhibit tumor growth to a certain extent (Figure 7A-7C), accompanied by increased apoptosis of GC cells (Figure 7D). Furthermore, there were significant difference between the groups with inhibition of serine (PHGDH) and asparagine (ASNS) synthesis, but neither inhibitor changed the chemotaxis ability of neutrophils (Supplementary Figure 10C). Taken together, these findings strongly illustrated that inhibition of serine (PHGDH) and asparagine (ASNS) synthesis in neutrophils leads to increased apoptosis of GC cells.

### 3.6. The NOX4^low^ CD66b^low^ signature predicts survival benefit from PD-1/PD-L1 inhibitor therapy

Having confirmed a positive correlation between NOX4 expression and neutrophil infiltration, we next investigated its clinical relevance to immunotherapy. An initial analysis of 16 GC patients from a neoadjuvant trial (NCT04208347) suggested that high NOX4/CD66b levels were associated with poorer response (Figure 7E and Supplementary Table 6). To definitively validate this, we leveraged our broader transcriptomic cohort, identifying an additional 43 patients who received PD-1/PD-L1 inhibitors for recurrent disease. Analysis of this expanded, homogeneous cohort of 59 patients revealed that the NOX4^low^ CD66b^low^ signature was a powerful predictor of long-term survival benefit (Log-rank p<0.001; Figure 7F and Supplementary Table 7-8), independent of other clinical variables (HR =0.372, 95% CI: 0.166- 0.676, p<0.001). Thus, starting from a biological correlation, we have identified a composite biomarker with validated clinical utility for predicting immunotherapy outcomes.

## 4. Discussion

Oxidative stress is one of the key factors affecting the occurrence and development of cancer. Excessive ROS can damage cellular proteins, lipids, and DNA, leading to lethal cell damage that has been implicated in a variety of pathologies, including cancer 20, 21. NOX4, as a member of the NOX family, can cause metabolic reprogramming and chemotherapy resistance in tumor cells by producing large amounts of ROS 22. ROS can also regulate the tumor microenvironment by affecting the immune response of various stromal cells to the tumor 20. Recent advances have highlighted that tumor-derived NOX4 regulates tumor stromal cells through a variety of molecular mechanisms and thus exerts a profound influence on tumor malignancy 10, 23. However, the correlation between tumor-derived NOX4 and neutrophils remains to be elucidated.

In the present study, we demonstrated that NOX4 was up-regulated in GC and that high expression of NOX4 was correlated with increased neutrophils infiltration and reduced overall survival in patients. Mechanistically, tumor-derived NOX4 appears to recruit neutrophils through GM-CSF and leads to increased amino acid synthesis in neutrophils. In line with our initial observation from the neoadjuvant cohort, subsequent analysis of a broader immunotherapy cohort (n=59) confirmed that the absence of the NOX4low CD66blow signature—reflecting a state of high NOX4 and high neutrophil infiltration—robustly predicts inferior clinical outcomes following PD-1/PD-L1 inhibitor therapy. To our knowledge, this is the first demonstration of a statistically significant correlation between NOX4 expression and neutrophil infiltration in human tumors; it is also the first demonstration of the role of tumor-derived NOX4 in the recruitment of neutrophils and induction of an immunosuppressive phenotype in the tumor microenvironment.

The role of neutrophils in tumors has attracted attention due to a common clinical phenomenon: leukemoid reaction (LKR) or tumor-related leukocytosis (TRL). This phenomenon is characterized by a significant increase in peripheral blood white blood cells (usually greater than 50×10^9^/L) with one or more naive cells as the main feature but no leukemic cell infiltration in any organ or tissue on clinical and pathological examination 24. Subsequently, a large number of studies have confirmed that patients with LKR or TRL have a significantly poorer prognosis 25-27. Mechanistic studies have found that leukocytosis in such patients may be due to the mass production and release of granulocyte colony-stimulating factor (G-CSF) by tumor cells to stimulate bone marrow hematopoiesis 28. Tumor-activated neutrophils in GC foster immune suppression and disease progression through the GM-CSF-PD-L1 pathway 15. The secretion of GM-CSF by HCC could stimulate the release of hepatocyte growth factor (HGF) from neutrophils, thereby promoting the growth and metastasis of tumor cells 29. Consistently, GC cells secrete G-CSF, which activates the JAK-STAT3 pathway of neutrophils, thus inducing and regulating the expression of PD-L2 in neutrophils and thereby mediating their transformation into an immunosuppressive phenotype 16. Here, we have significantly expanded upon these previous observations. Our findings confirm that GC cell-derived NOX4 can induce neutrophil recruitment through secretion of GM-CSF, resulting in up-regulation of PD-L1 and Arg-1 expression, which manifests as an immunosuppressive phenotype. Although beyond the scope of this clinical-correlative study, future work is needed to unravel the precise mechanism of NOX4-mediated GM-CSF upregulation, a finding that would provide a more complete understanding of the biology described here.

McAvoy *et al*. found that during Gram-negative sepsis and endotoxemia, CD14 is essential for neutrophils recruitment within the liver microcirculation 30. Although numerous studies have shown that CD14 can be highly expressed in various tumors 31-33, including gastric cancer 34, its correlation with neutrophils has not been reported in cancers. DKK-1 suppresses PTGS2-induced macrophage and neutrophils recruitment in lung metastases by antagonizing breast cancer cell non-canonical WNT/PCP-RAC1-JNK signaling 35. However, in our *in vivo* experiments, we did not find that it can recruit neutrophils into the tumor microenvironment. The inconsistencies *in vivo* and* in vitro* experiments were considered to be related to the influence of other immune cells in the tumor microenvironment and subtypes of tumor associated neutrophils.

Numerous studies from mouse models have confirmed that tumor infiltrating neutrophils have dual effects on promoting and inhibiting tumors 36. Using single-cell RNA sequencing, Xue *et al*. analyzed the cellular landscape of 189 liver cancer patients and mouse model samples, revealing the heterogeneity of tumor associated neutrophils for the first time, and discovering and validating the tumor promoting mechanisms of two key subtypes, CCL4+and PD-L1+TAN 37. When neutrophils with Sellhi (including N1a, N1b, and N2) phenotype were increased in human lung cancer tumors, the patient is more likely to benefit from immunotherapy 38.

As early as 2011, "tumor inflammation" and "immune evasion" were included in the top 10 hallmarks of cancer 39. Whenever inflammation, infection, or injury occurs, neutrophils are the first to respond. The neutrophil is the most abundant leukocyte in the body, and it plays a crucial role in promoting angiogenesis, inhibiting immunity, and contributing to cancer progression through a variety of mechanisms 40-42. Molecularly, several studies have shown that neutrophil extracellular traps (NETs) produced by tumor-associated neutrophils (TANs) can degrade the extracellular matrix and promote cancer cell extravasation and metastasis 43-45. Moreover, neutrophils promote tumor growth by releasing ROS, which in turn induce DNA damage 46. In this study, we found that the infiltration of neutrophils in GC tissues was significantly higher than that in adjacent gastric tissue. Furthermore, *in vitro* and *in vivo* studies also confirmed that the tumor-infiltrating neutrophils promoted tumor progression by inhibiting GC cell apoptosis. These results highlight the vital role of neutrophils in promoting tumor progression.

Accumulating evidence has demonstrated that the remodeling of amino acid metabolism has a significant impact on tumor cells and the tumor immune microenvironment 47, 48. In particular, the metabolic state of tumor-associated neutrophils (TANs) is a critical determinant of their pro-tumor functions. For instance, TANs in lung adenocarcinoma models upregulate Glut1 and glucose metabolism 49, while in ovarian cancer models, they utilize glutaminolysis to fuel OXPHOS and support immunosuppression 50. A classic mechanism involves TANs secreting Arg-1 to deplete L-arginine and suppress T-cell function 51, 52. In line with these concepts, our study reveals that tumor-derived NOX4 drives a specific metabolic reprogramming in neutrophils, characterized by the upregulation of phosphoglycerate dehydrogenase (PHGDH) and asparagine synthetase (ASNS).

The significance of this reprogramming likely extends beyond mere energy production. PHGDH, a key enzyme in the serine synthesis pathway, is often hijacked by tumor cells to support their growth and evade apoptosis 53, 54. In neutrophils, we propose that PHGDH-derived serine is crucial for synthesizing key proteins and signaling molecules necessary for their sustained immunosuppressive activity. Similarly, ASNS, which catalyzes asparagine synthesis from aspartate and glutamine, is a known facilitator of tumor cell proliferation 55, with its inhibition being a validated anticancer strategy 56. Within neutrophils, the NOX4-driven upregulation of ASNS may provide the asparagine critical for maintaining their functional longevity and the robust protein synthesis required for cytokine production in the demanding tumor microenvironment. Critically, we posit that inhibiting PHGDH or ASNS does not simply exert a toxic effect that indiscriminately kills neutrophils. Instead, it specifically disrupts the metabolic infrastructure that enables their pro-tumor functions. By depriving them of serine and asparagine, key building blocks for functional execution, such inhibition would cripple their capacity to protect cancer cells, ultimately leading to the observed increase in tumor cell apoptosis. This model shifts the focus from general neutrophil viability to the metabolic dependencies of their functional state. In conclusion, our findings position NOX4 as a master regulator that metabolically primes neutrophils towards a pro-tumor phenotype via PHGDH and ASNS. Targeting this specific metabolic axis in TANs presents a promising strategy to disarm their tumor-promoting capacity without necessarily eradicating them, potentially overcoming the limitations of current therapies.

Numerous studies have focused on anti-PD-1 therapy (carelizumab) combined with neoadjuvant chemotherapy for the treatment of GC 57, 58, and the results have confirmed that this therapeutic strategy can significantly improve the rate of tumor regression and the rate of pathological complete response (pCR) in patients. Given the higher NOX4 expression and neutrophil infiltration in GC and the resulting immunosuppressive microenvironment, stratification of patients according to NOX4 expression may better identify the population that is likely to benefit from carelizumab. Our study not only describes the interplay between NOX4 and neutrophils in the TME but, crucially, links this biology to a clinically actionable outcome. The most significant finding in this context is that the NOX4^low^CD66b^low^ signature emerged as a strong and independent predictor of survival in a dedicated cohort of 59 patients treated with PD-1/PD-L1 inhibitors. This moves beyond correlation and provides direct evidence for its role in modulating therapy response. We postulate that tumors with low NOX4 and low neutrophil infiltration represent an immune contexture less dominated by myeloid-derived suppression, thereby potentially allowing for a more robust T-cell response upon PD-1 blockade. This signature therefore holds translational potential as a novel composite biomarker to help identify GC patients most likely to derive long-term benefit from immunotherapy.

## 5. Conclusions

Based on our *in vitro* and *in vivo* results, we propose a model related to increasing immunosuppression within the GC tumor microenvironment (Figure 7G). First, in GC cells, ROS accumulation caused by high NOX4 expression leads to increased secretion of GM-CSF through various mechanisms. Second, released GM-CSF recruits and polarizes neutrophils, which is accompanied by the induction of PD-L1 expression in neutrophils. Third, these activated neutrophils have increased synthesis of serine and asparagine and thus exert a protumor effect by inhibiting the apoptosis of GC cells. In this way, tumor-derived NOX4 appears to contribute to tumor progression by inducing neutrophil activation and fostering immune suppression in GC. In the future, combinational therapeutic strategies aimed at interfering with NOX4 expression and/or these pathological neutrophils may be developed to provide novel strategies for GC treatment.

## Supplementary Material

Supplementary figures and tables.

## Data Availability

The datasets used/or analyzed during the study are available from the corresponding author on reasonable request.

## Abbreviations

GC: gastric cancer
NOX4: NADPH oxidase 4
GM-CSF: granulocyte-macrophage colony-stimulating factor
ROS: reactive oxygen species
PD-L1: programmed cell death receptor-ligand 1
Arg-1: arginase I
PHGD: phosphoglycerate dehydrogenase
ASNS: asparagine synthetase
PLA2G3: phospholipase A2: group III
TAMs: tumor-associated macrophages
CAFs: cancer associated fibroblasts
NKs: natural killer cells
qRT-PCR: quantitative real-time polymerase chain reaction
WB: Western blot
GEPIA: Gene Expression Profiling Interactive Analysis
HUVECs: Human umbilical vein endothelial cells
FBS: fetal bovine serum
PVDF: polyvinylidene fluoride
HRP: horseradish peroxidase
NOD/SCID: non-obese diabetic severe combined immunodeficiency
TRL: tumor-related leukocytosis
KEGG: Kyoto Encyclopedia of Genes and Genomes
LKR: Leukaemoid reaction
G-CSF: granulocyte- colony stimulating factor
NETs: Neutrophil Extracellular Traps
TRG: tumor regression grade
