# Supplementary material for: The Role of NADPH Oxidase 4 in Neutrophil-Mediated Immune Escape in Gastric Cancer
Source: Int J Biol Sci. 2026 May 1;22(9):4919–37. doi: 10.7150/ijbs.121960 (PMC13182548; doi:10.7150/ijbs.121960)
Supplement: Supplementary file 1 — Supplementary figures and tables. [file ijbsv22p4919s1.pdf]

Supplementary Tables and Figures

Supplementary Table 1. Specific primers for quantitative real-time PCR

Gene name		Sequences (5’-3’)
NOX4	F	GGACCTTTGTGCCTGTACTGTG
	R	GTGAGGGATGACTTATGACCGA
CREB3L3	F	ATGAATACGGATTTAGCTGCTGG
	R	AGGAAGTCGTCAGAGTCGGG
GGT1	F	CTGGGGAGATCCGAGGCTAT
	R	GATGACGGTCCGCTTGTTTTC
SNPH	F	CCAGGAAGTAGACGGACCTCT
	R	CTGCCCTTGTAGGAGCCAG
BIK	F	GACCTGGACCCTATGGAGGAC
	R	CCTCAGTCTGGTCGTAGATGA
RGPD5	F	GCCGTTGAATGTTACAGGCG
	R	CTGCTCTTTCGACCCAGTATTT
TLDC2	F	GGAGGAGGGTAACGAAGAGGA
	R	TGAAACCGTCCCTTGACGTG
MTHFD2	F	CTGCGACTTCTCTAATGTCTGC
	R	CTCGCCAACCAGGATCACA
PKC2	F	GCCATCATGCCGTAGCATC
	R	AGCCTCAGTTCCATCACAGAT

ASNS	F	GGAAGACAGCCCCGATTTACT
	R	AGCACGAACTGTTGTAATGTCA
PHGDH	F	CTGCGGAAAGTGCTCATCAGT
	R	TGGCAGAGCGAACAATAAGGC
AARS1	F	TCCGGCAGCGATTTATAGATTTC
	R	GCCTGCATTGGCAAAGAGC
PLA2G3	F	TGTGGAGTTGGAGATTCTGCT
	R	CGGTAGTTTCGGATGCCATAGTT
MSMO1	F	TGCTTTGGTTGTGCAGTCATT
	R	GGATGTGCATATTCAGCTTCCA
SALL4	F	AGCACATCAACTCGGAGGAG
	R	CATTCCCTGGGTGGTTCACTG
CHRD	F	TTCGGCGGGAAGGTCTATG
	R	ACTCTGGTTTGATGTTCTTGCAG
WFDC1	F	GCTACAACGGATGCGCCTA
	R	CAAGCCATCGAGGTTTCGG

Supplementary Table 2. Antibodies used for Western blot, immunohistochemistry and immunofluorescence

Antibody	catalog	Dilution	Source
**Western blot**			
Anti-NOX4	ab133303	1:1000	Abcam
Anti-PD-L1	ab205921	1:100	Abcam
Anti-Arg1	TP71241S	1:200	Abmart
Anti-Bcl-2	T40056S	1:1000	Abmart
Anti-Bcl-XL	T55050S	1:1000	Abmart
Anti-Bax	T40051S	1:1000	Abmart
Anti-Caspase-8	T40045S	1:1000	Abmart
Anti-Caspase-9	T40046S	1:1000	Abmart
Anti-GAPDH	G9545	1:10000	Sigma
Secondary antibody	HRP conjugated goat anti-rabbit IgG	1:3000	Sigma
Secondary antibody	HRP conjugated goat anti-mouse IgG	1:3000	Sigma
**Immunohistochemistry**			
Anti-NOX4	ab133303	1:200	Abcam
Anti-CD66b	ab214175	1:200	Abcam
Secondary antibody	HRP conjugated goat anti-rabbit IgG	1:3000	Sigma
Secondary antibody	HRP conjugated goat anti-mouse IgG	1:3000	Sigma
**Immunofluorescence**			
Anti-NOX4	ab133303	1:500	Abcam

Anti-PD-L1	ab205921	1:200	Abcam
Anti-CD66b	ab214175	1:100	Abcam
Anti-CD11b	Ab8878	1:200	Abcam
Anti-Ly6G	T61229S	1:200	Abmart
Secondary antibody	Alexa Fluor 546 anti-rabbit IgG	1:1000	Invitrogen
Secondary antibody	Alexa Fluor 488 anti-mouse IgG	1:1000	Invitrogen

Supplementary Table 3. Antibodies used for flow cytometry analysis

Antibody	catalog	Dilution	Source
Ms CD45 APC-Cy7 30-F11	557659	1:200	BD
APC anti-human CD66b	305118	1:100	Biolegend
CD11b BB700 M1/70	566416	1:200	BD
Ms Ly-6G PE-Cy7 1A8	560601	1:200	BD
Ms CD45R Horizon V500 RA3-6B2	561226	1:200	BD
Ms CD49b PE HM Alp2	558759	1:200	BD
Ms F4/80 BV421 T45-2342	565411	1:150	BD
Ms CD86 BV605 GL1	563055	1:200	BD
Ms CD11c APC-R700 N418	565872	1:200	BD
Ms Ly-6C APC AL-21	560595	1:200	BD
Ms NK1.1 Alexa 700 PK136	560515	1:100	BD
BB700 Rat IgG2a, κ Isotype Control	566413	1:500	BD
Ms I-A/I-E BB515 2G9	565254	1:200	BD
Rat IgG2a Kpa ItCl BB515 R35-95	564418	1:200	BD
Rat IgM Kpa ItCl APC R4-22	551486	1:200	BD
Rat IgG2a Kpa ItCl PE-Cy7 R35-95	552784	1:500	BD
Rabbit monoclonal to PD-L1	ab205921	1:100	Abcam
CD45-APC	561487	1:200	BD
Donkey anti-Rabbit IgG(H+L)	A10042	1:500	Thermofisher

Supplementary Table 4. Correlation between expression of NOX4 and clinic-pathological features of GC patients

Variable	Cases（%）	NOX4 expression		P value
		Low（n=84）	High（n=119）	
**Age**				
≤ 60	96（47.3）	41	55	0.716
> 60	107（52.7）	43	64	
**Gender**				
Male	99（48.8）	44	55	0.387
Female	104（51.2）	40	64	
**TNM Stage**				
I+II	62（30.5）	33	29	0.023*
III+ IV	141（69.5）	51	90	
**T Stage**				
T1+ T2	33（16.3）	17	16	0.196
T3+ T4	170（83.7）	67	103	
**N Stage**				
N0+ N1	12（1.5）	9	3	0.015*
N2+ N3	191（38.9）	75	116	
**M Stage**				
M0	198（97.5）	81	117	0.392
M1	5（2.5）	3	2	
**Tumor Size**				
＜5cm	57（28.1）	30	27	0.042*
≥5cm	146（71.9）	54	92	
**Venous Invasion**				
No	82（40.4）	44	38	0.003**
Yes	121（59.6）	40	81	
**Nerve Invasion**				
No	98（48.3）	44	54	0.325
Yes	105（51.7）	40	65	
**Histologic Grade**				
Well/Moderate	53（26.1）	32	21	0.001**
Poor	150（73.9）	52	98	

Abbreviations: *P<0.05, **P<0.01.

Supplementary Table 5. Correlation between infiltration level of CD66b and clinic-pathological features of GC patients

Variable	Cases（%）	CD66b infiltration		P value
		Low（n=97）	High（n=106）	
**Age**				
≤ 60	96（47.3）	45	51	0.806
> 60	107（52.7）	52	55	
**Gender**				
Male	99（48.8）	53	46	0.109
Female	104（51.2）	44	60	
**TNM Stage**				
I+II	62（30.5）	37	25	0.024*
III+ IV	141（69.5）	60	81	
**T Stage**				
T1+ T2	33（16.3）	15	18	0.991
T3+ T4	170（83.7）	82	98	
**N Stage**				
N0+ N1	12（1.5）	6	6	0.874
N2+ N3	191（38.9）	91	100	
**M Stage**				
M0	198（97.5）	97	101	0.030*
M1	5（2.5）	0	5	
**Tumor Size**				
＜5cm	57（28.1）	34	23	0.034*
≥5cm	146（71.9）	63	83	
**Venous Invasion**				
No	82（40.4）	39	43	0.958
Yes	121（59.6）	58	63	
**Nerve Invasion**				
No	98（48.3）	47	51	0.961
Yes	105（51.7）	50	55	
**Pathologic Differentiation**				
Well/Moderate	53（26.1）	26	27	0.829
Poor	150（73.9）	71	79	
**NOX4 expression**				
Low	84（41.4）	48	36	0.025*
High	119（58.5）	49	70	

Abbreviations: *P<0.05, **P<0.01.

Supplementary Table 6. Baseline and treatment response of 16 patients with GC who received neoadjuvant therapy

Variable	chemotherapy + camrelizumab (n=8)	Chemotherapy (n=8)	P value
**Age**	58.6	60.2	/
**NOX4 expression**			
High	5 (62.5%)	6 (75%)	0.054
Low	3 (37.5%)	2 (25%)	
**CD66b infiltration (16 cells/HP)**			
High	4 (50%)	5 (62.5%)	0.077
Low	4 (50%)	3 (37.5%)	
**Treatment response**			
Responder	6 (75%)	4 (50%)	<0.001**
Non-responder	2 (25%)	4 (50%)	

Abbreviations: *P<0.05, **P<0.01.

Supplementary Table 7. Correlation between patients who received immunotherapy and clinic-pathological features of GC patients

Variable	Cases（%） (n=59)	$NOX4^{low}CD66b^{low}$ (n=15)	$NOX4^{High}CD66b^{High}$ (n=23)	Others* (n=21)	P value
**Age**					
≤ 60	26 (40%)	6	11	9	0.171
> 60	33 (60%)	9	12	12	
**Gender**					
Male	35 (59%)	8	14	13	0.223
Female	24 (41%)	7	9	8	
**PD-L1 expression**					
CPS<1	43 (63%)	10	17	16	0.446
CPS≥1	16 (27%)	5	6	5	

**Treatment response**					
Responder	33 (56%)	10	10	13	0.473
Non-responder	26 (44%)	5	13	8	

Abbreviations: Others refers to $NOX4^{high}CD66^{low}$ and $NOX4^{low}CD66^{high}$. *P<0.05, **P<0.01.

Supplementary Table 8. Univariate and Multivariate Analyses of Variables Associated with OS of GC Patients who received immunotherapy

	OS					
	Univariate			Multivariate		
**Variables**	**HR**	**95% CI**	**P Value**	**HR**	**95% CI**	**P Value**
Age（＞60 vs ≤60）	1.037	0.712-1.407	0.703			NA
Gender（Female vs Male）	1.141	0.798-1.512	0.422			NA
PD-L1 expression（CPS<1 vs CPS≥1）	0.628	0.517-1.016	0.043	0.762	0.603-1.270	NA
Treatment response（Responder vs Non-responder）	0.598	0.349-1.014	0.056			NA
NOX4 and CD66b expression（All low vs Other）	0.425	0.266-0.801	＜**0.00****	0.372	0.176-0.676	＜**0.00****

Abbreviations: OS, overall survival; HR, hazard ratio; CI, confidential interval; NA, not adopted. Boldface type indicates significant values. *P<0.05, **P<0.01.

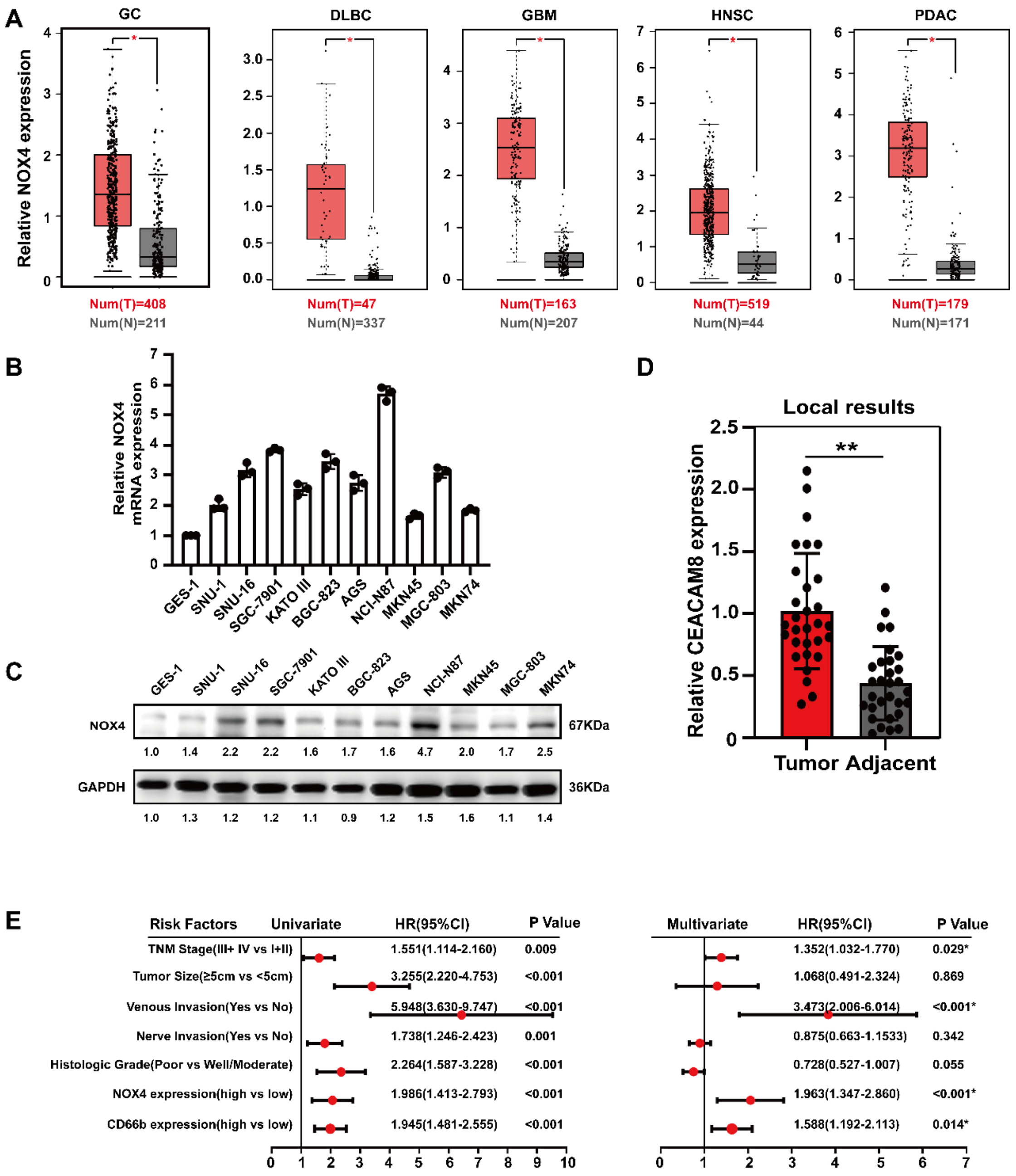

Supplementary Figure 1. NOX4 expression is up-regulated in cancers and correlated with poor prognosis in GC. (A) The expression of NOX4 in tumor tissues compared with corresponding adjacent tissues was analyzed using GEPIA datasets. (B) mRNA expressions of NOX4 in normal gastric mucosal cells (GES-1) and 10 gastric cancer cell lines. (C) Western blot analysis of NOX4 expression in normal gastric mucosal cells (GES-1) and 10 gastric cancer cell lines. (D) Relative mRNA expression levels of *CEACAM8* (CD66b) in 30 pairs of human GC, as determined by qRT-PCR. Expression levels are presented as 2^–ΔCt. (E) Univariate and multivariate

COX proportional hazard analysis of factors associated with overall survival in 203 GC patients. The data represent three independent experiments. Data are presented as the mean ± SD. *P < 0.05; **P < 0.01; ***P< 0.001.

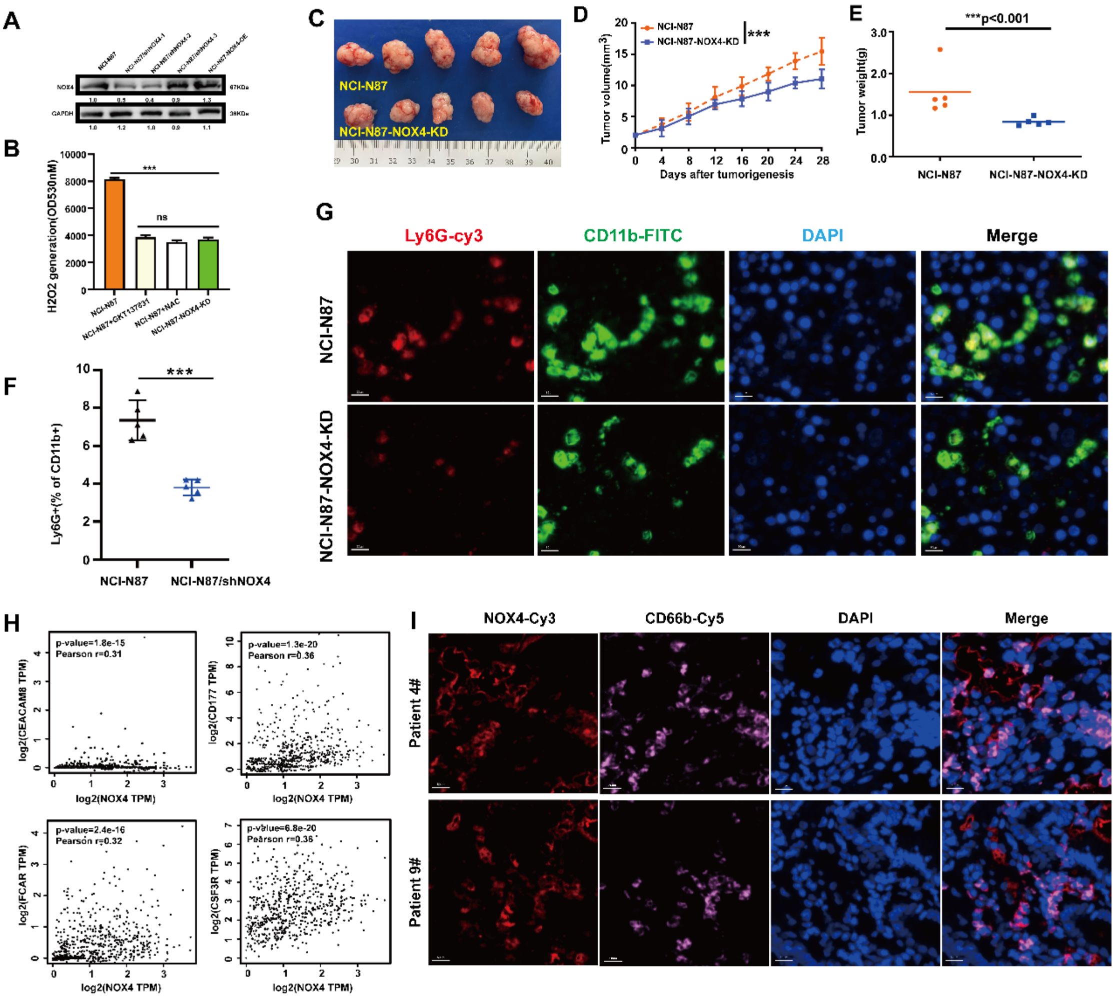

Supplementary Figure 2. Tumor-derived NOX4 promotes intratumoral infiltration of neutrophils. (A) Western blot analysis of NOX4 expression in NCI-N87 cell line. (B) Production of H2O2 of NCI-N87 cells treated with GKT137831 (20μM) or NAC (2.5mM). (C-E) Tumor growth curves and tumor burdens in immunodeficient nude mice injected subcutaneously with NCI-N87, and NCI-N87-NOX4-KD cells (n=5). (F) Quantification of tumor-infiltrating neutrophil analyzed by flow cytometry on NCI-N87 and NCI-N87/shNOX4 GC cells tumors grafted into BALB/C nude mice. (G) Quantification of tumor-infiltrating neutrophils analyzed by Immunofluorescence

staining using the surface markers of CD11b and Ly6G on NCI-N87 and NCI-N87-NOX4-KD GC cells tumors grafted into BALB/C nude mice. (H) Correlation between NOX4 expression and a panel of neutrophil marker genes in the TCGA-STAD cohort. (I) Immunofluorescence double staining revealed high co-expression of NOX4 and CD66 in two patients. The data represent three independent experiments. Data are presented as the mean ± SD. *P < 0.05; **P < 0.01; ***P< 0.001.

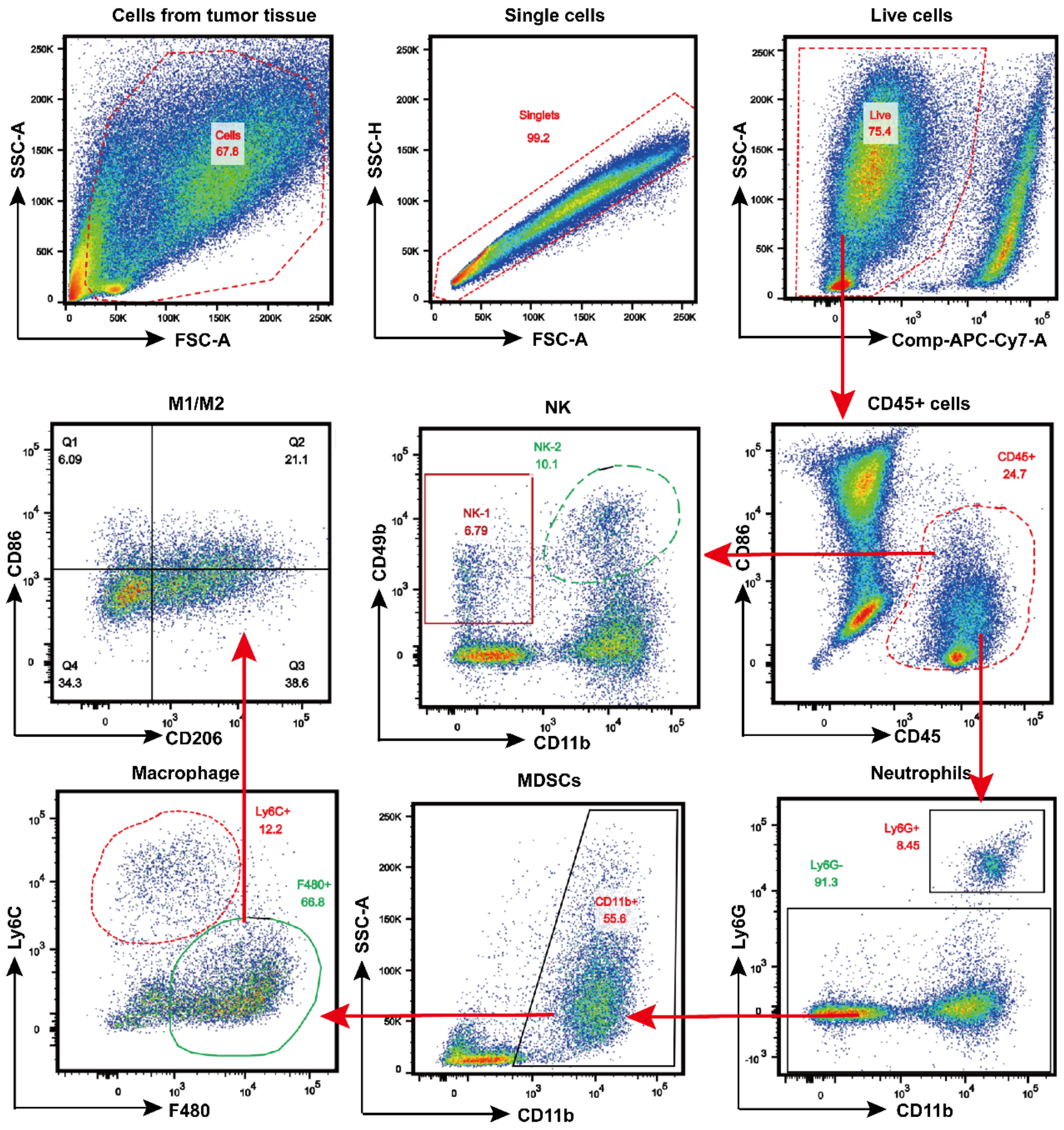

Supplementary Figure 3. Gating strategy for flow cytometry analysis of

immune cells in GC tumors. The representative dot plots of flow cytometry staining of natural killer cells (CD49b$^+$CD11b$^+$), macrophages (Ly6C$^+$F480$^+$), macrophages M1 (F480$^+$CD86$^+$), macrophages M2 (F480$^+$CD206$^+$), myeloid-derived suppressor cells (MDSCs; CD11b$^+$) and neutrophils(CD11b$^+$Ly6G$^+$).

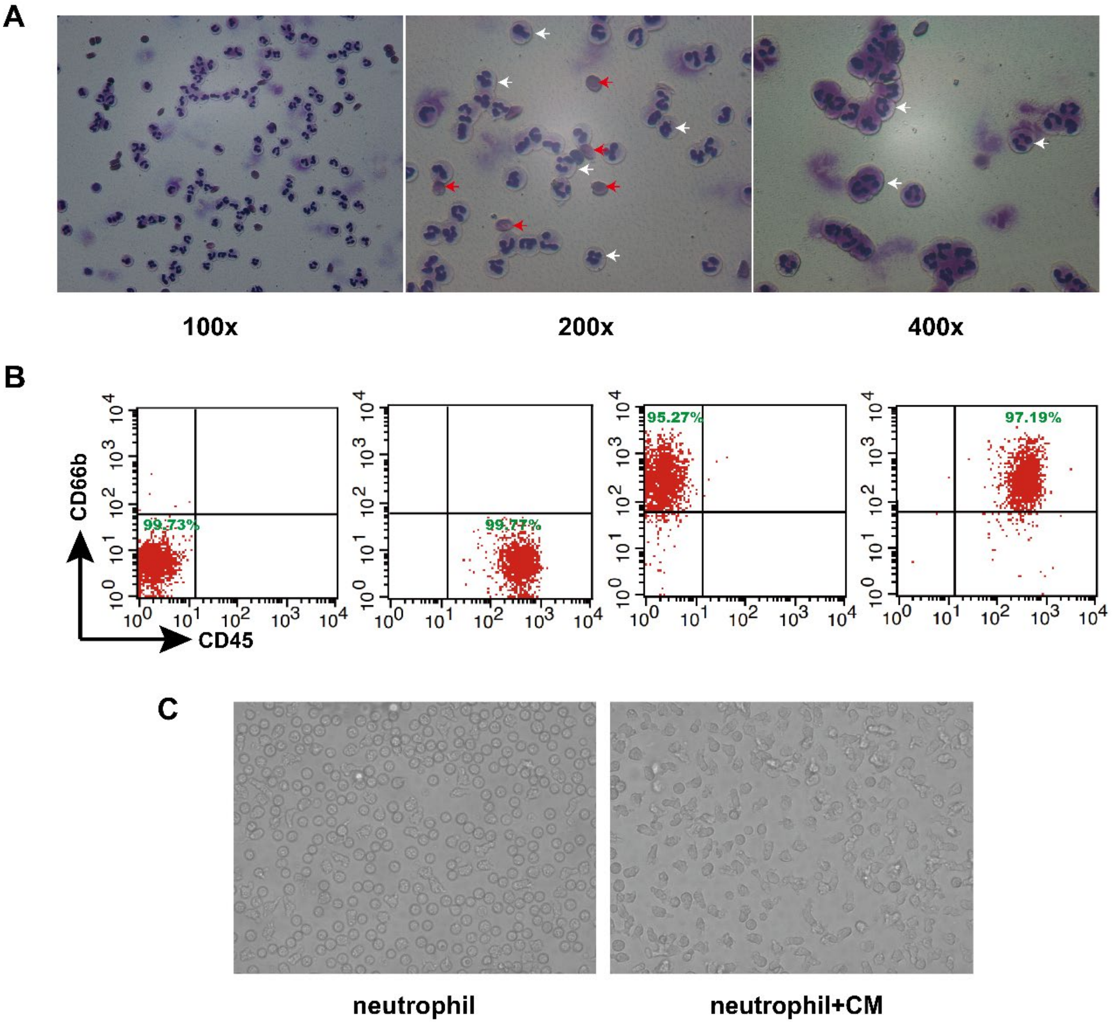

Supplementary Figure 4. Purification and identification of neutrophils. (A, B) Purification of neutrophils detected by Diff staining and flow cytometry. (C) Morphology of non-activated and activated neutrophils. Data are presented as the mean ± SD. *P < 0.05; **P < 0.01; ***P< 0.001.

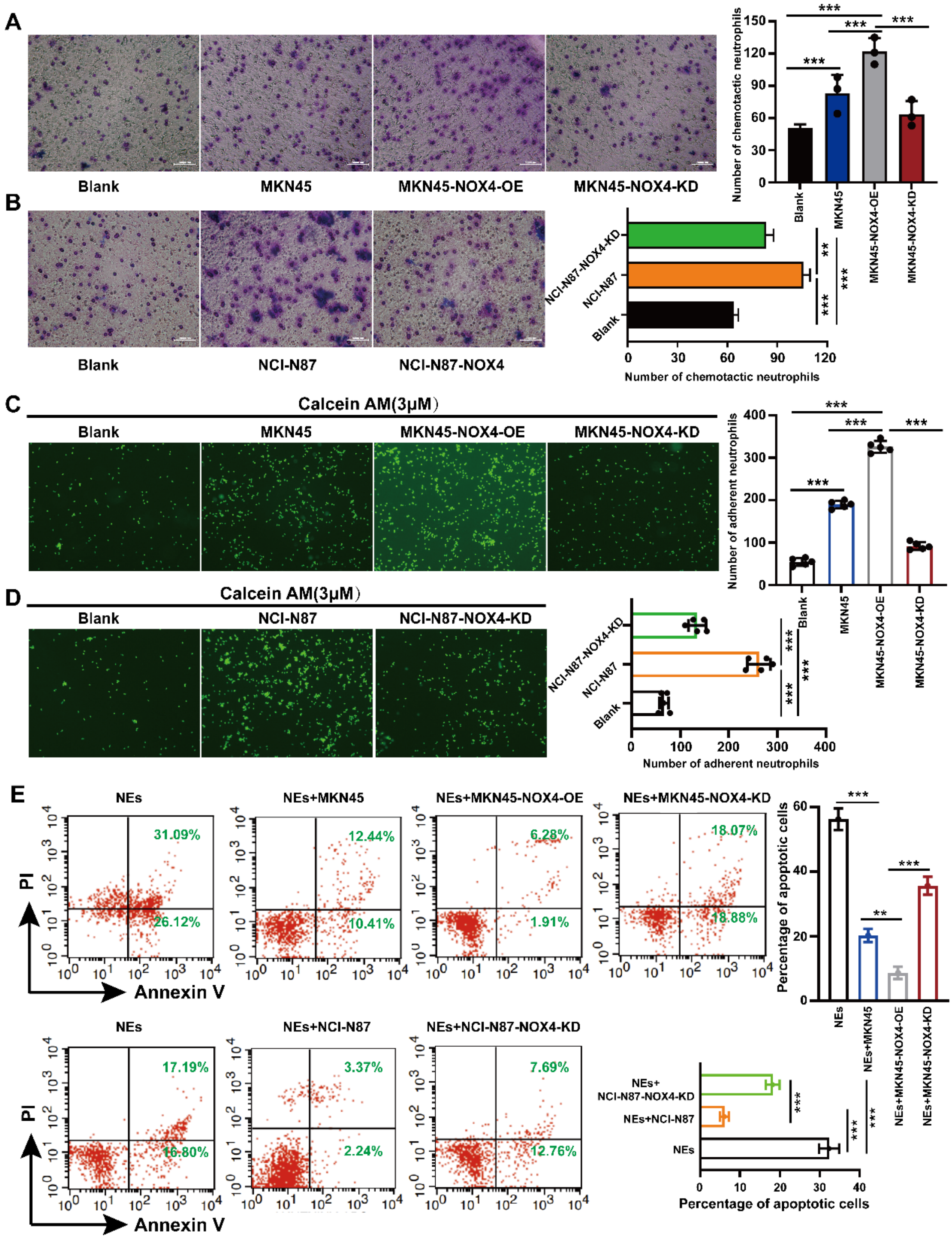

Supplementary Figure 5. Tumor-derived NOX4 conditioned neutrophils enhanced chemotaxis and adhesion capacity, inhibited apoptosis of gastric cancer cells. (A, B) Neutrophils were cultured with conditioned medium of MKN45, MKN45-NOX4-OE, MKN45-NOX4-KD, NCI-N87 and NCI-N87-NOX4-KD GC cells as indicated, chemotactic capacity were evaluated by the transwell assay. (C, D)Tumor-

derived NOX4 conditioned neutrophils were labeled with Calcein AM (3μM) and co-cultured with HUVECs as indicated, representative images of adherent neutrophils and statistics analysis. (E) The effect of tumor-derived NOX4 conditioned neutrophils on KNK45 and NCI-N87 GC cell apoptosis were assessed by flow cytometry analysis. The data represent three independent experiments. Data are presented as the mean ± SD. *P < 0.05; **P < 0.01; ***P< 0.001.

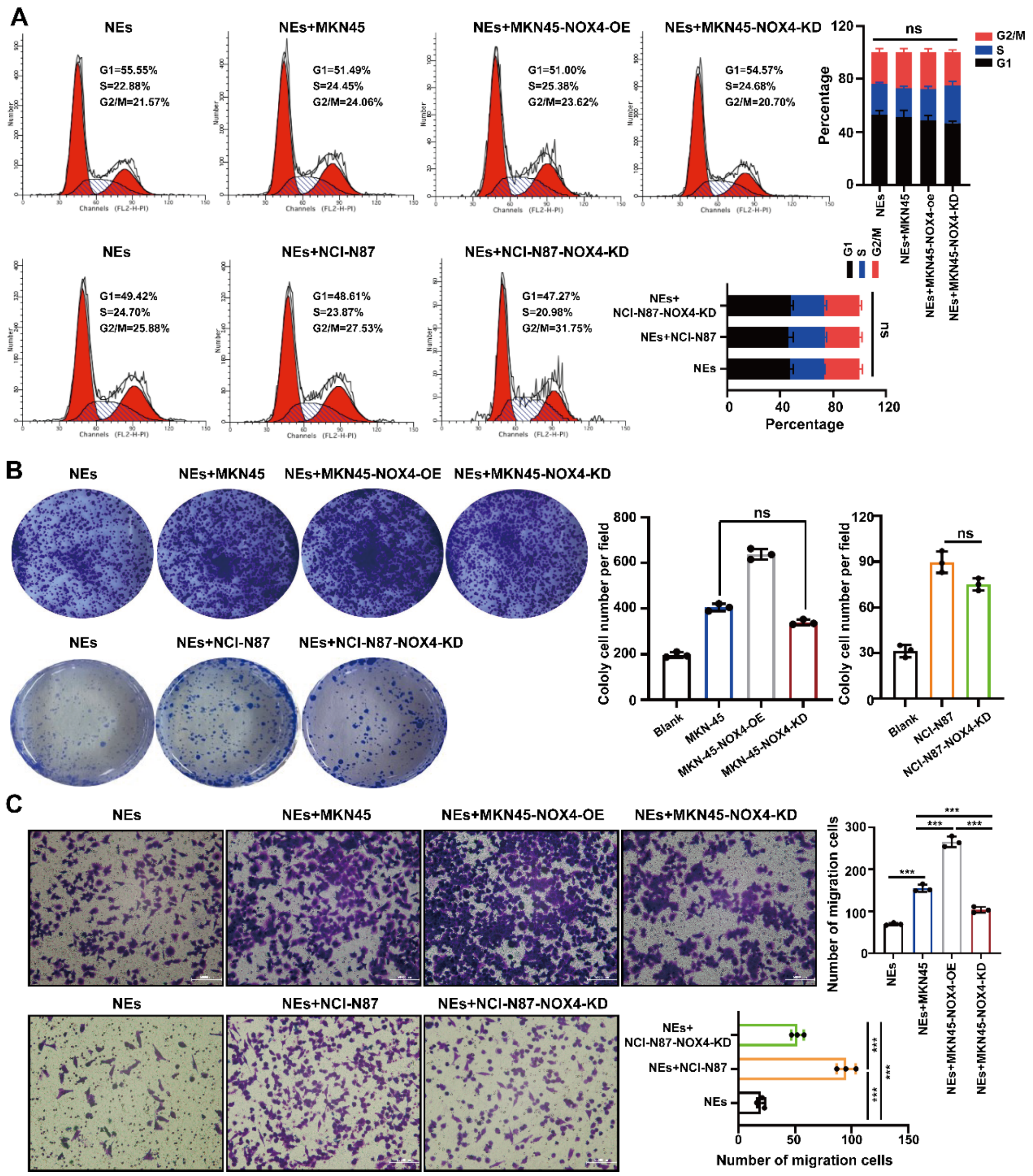

Supplementary Figure 6. The effect of activated neutrophils on GC cells.

(A) The cell cycle distribution and statistics analysis of KNK45 and NCI-N87 GC cells co-cultured with tumor-derived NOX4 conditioned neutrophils were analyzed by flow cytometry. (B) The effect of tumor-derived NOX4 conditioned neutrophils on KNK45 and NCI-N87 GC cell proliferation were assessed by colony formation assay. (C) Transwell assays were performed to assess the effect of tumor-derived NOX4 conditioned neutrophils on KNK45 and NCI-N87 GC cell migration. The data represent

three independent experiments. Data are presented as the mean ± SD. *P < 0.05; **P < 0.01; ***P< 0.001.

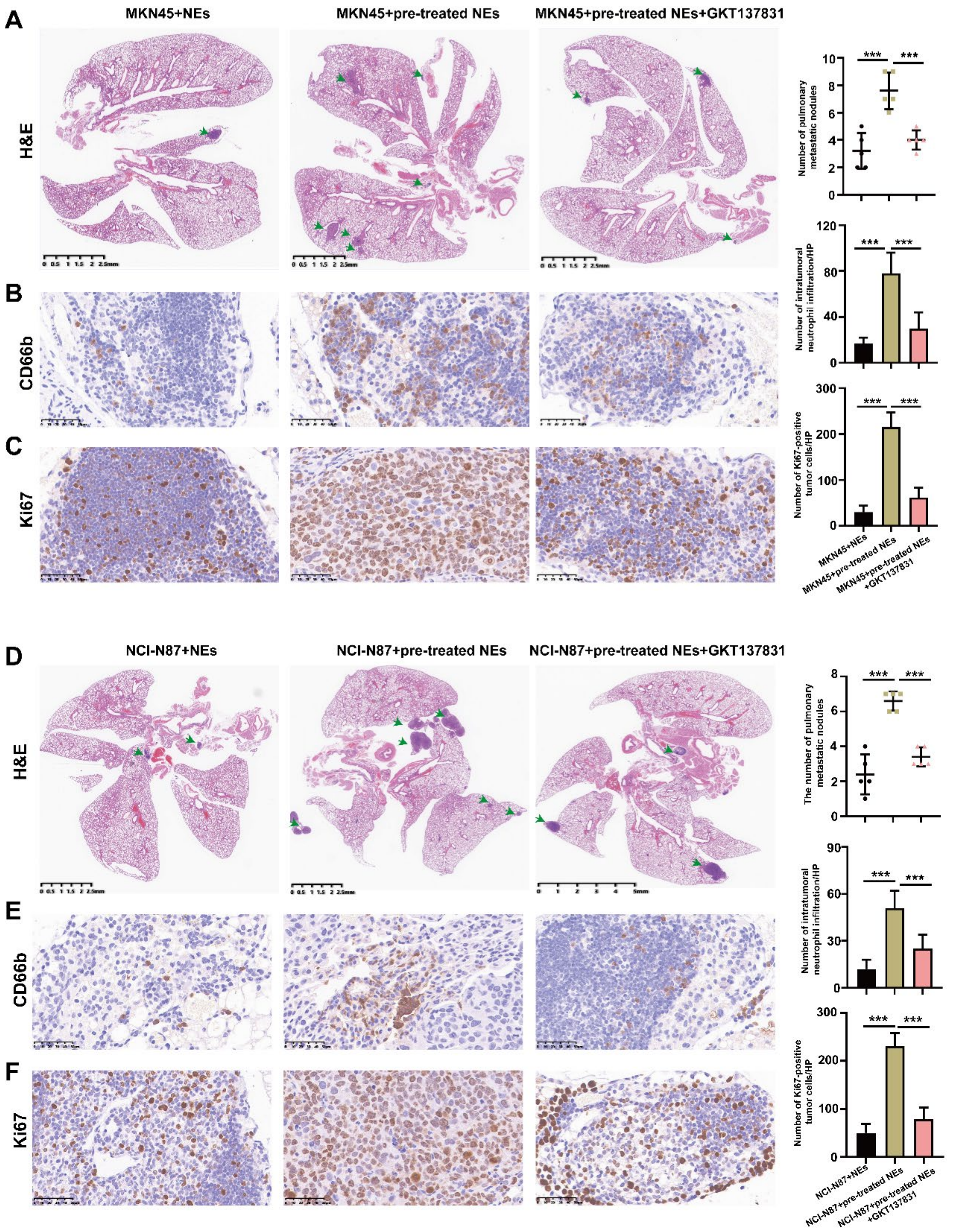

Supplementary Figure 7. Pro-metastatic role of tumor-derived NOX4 activated neutrophils in vivo. Gastric cancer cells were co-injected with either NOX4-activated neutrophils or control neutrophils into NOD/SCID mice via the tail vein

to establish the lung metastasis model. (A) MKN45 groups representative H&E staining of lung sections showing metastatic foci (n = 5 per group). Scale bar, 2.5mm or 5mm. (B) MKN45 groups immunohistochemical staining for CD66b (neutrophil marker) in metastatic lesions. Scale bar, 50 μm. (C) MKN45 groups immunohistochemical staining for Ki67 in metastatic lesions (n = 5 per group). Scale bar, 50 μm. (D) NCI-N87 groups representative H&E staining of lung sections showing metastatic foci (n = 5 per group). Scale bar, 2.5mm or 5mm. (E) NCI-N87 groups immunohistochemical staining for CD66b (neutrophil marker) in metastatic lesions. (n = 5 per group) (F) NCI-N87 groups immunohistochemical staining for Ki67 in metastatic lesions (n = 5 per group). Scale bar, 50 μm. Data are presented as mean ± SEM. *p < 0.05, **p < 0.01.

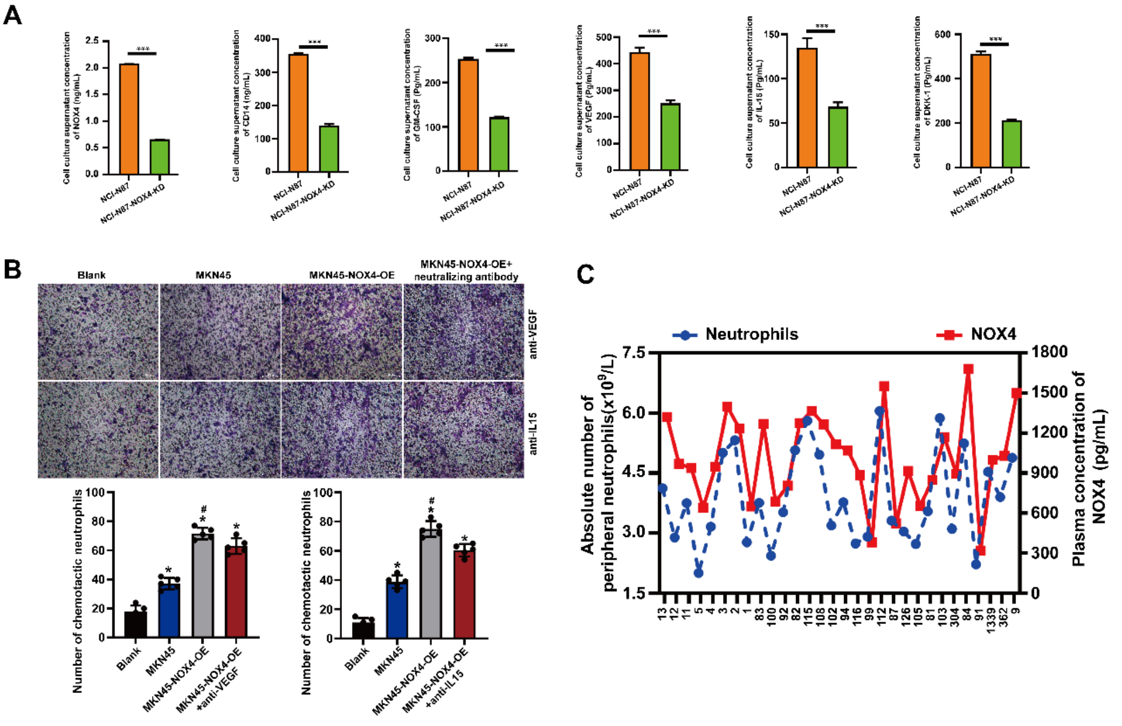

Supplementary Figure 8. Various cytokines are involved in the recruitment of neutrophils. (A) NOX4, CD14, GM-CSF, VEGF, IL-15 and DKK-1 were verified by ELISA in the co-culture supernatants of NCI-N87 and NCI-N87-NOX4-

KD cells and healthy donor neutrophils. (B) The conditioned medium of MKN45-NOX4-OE GC cell were treated with anti-VEGF (20μg/ml) or anti-IL-15 (20μg/ml), neutrophils chemotactic capacity were evaluated by the transwell assay. (C) The plasma NOX4 concentration and the absolute number of peripheral blood neutrophils in 30 patients with newly diagnosed GC. The data represent three independent experiments. Data are presented as the mean ± SD. *P < 0.05; **P < 0.01; ***P< 0.001.

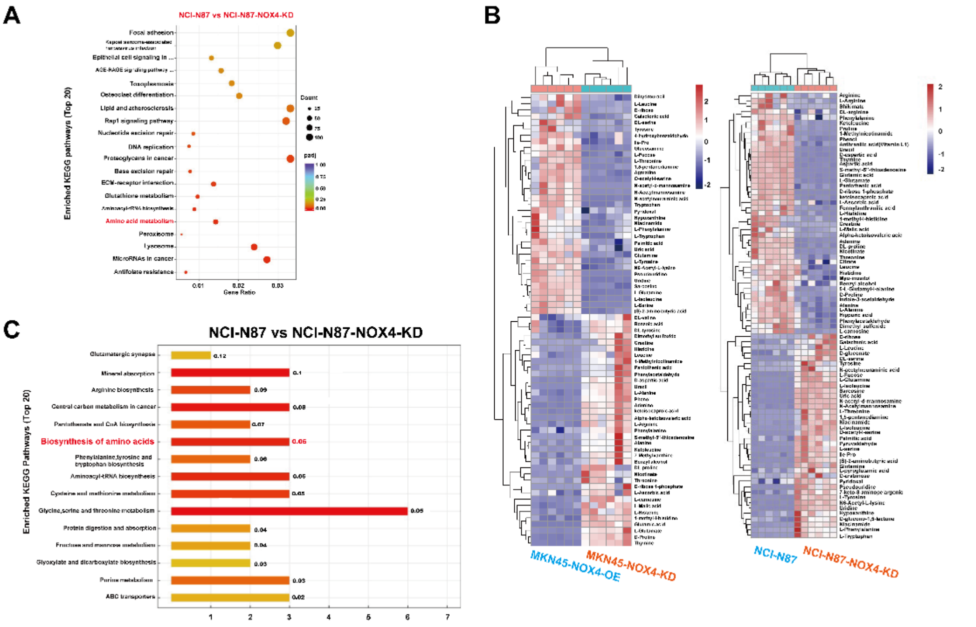

Supplementary Figure 9. Biosynthesis of amino acids pathway altered within neutrophils by tumor-derived NOX4 in GC. (A) KEGG pathway analysis of RNA-sequencing showed the significantly differences in biosynthesis of amino acids pathway of neutrophils in NCI-N87 vs NCI-N87-NOX4-KD group. (B) Heat map showed the metabolic differences in neutrophils conditioned by conditioned medium of MKN45-NOX4-KD, MKN45-NOX4-OE, NCI-N87 and NCI-N87-NOX4-KD GC cells. (C) KEGG pathway analysis of non-targeted metabolic mass spectrometry in neutrophils conditioned by conditioned medium of NCI-N87 and NCI-N87-NOX4-KD GC cells.

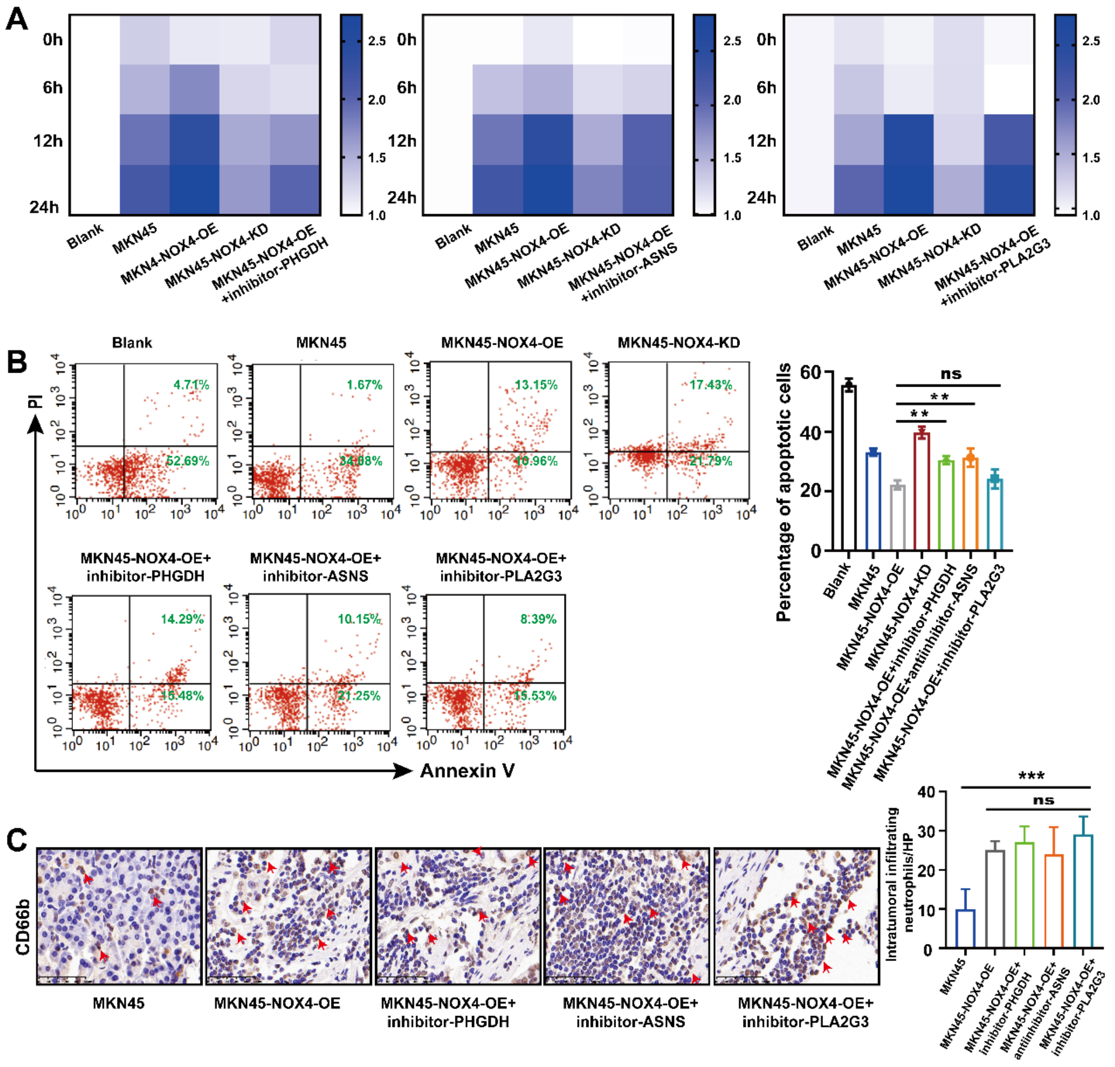

Supplementary Figure 10. Serine (PHGDH) and asparagine (ASNS) play vital role in neutrophil pro-tumor. (A) Heat map showed the viability of neutrophils conditioned by NOX4 high-expression medium with inhibitor of PHGDH （15μM）, inhibitor of ASNS（20μM） and inhibitor of PLA2G3（50μM）. (B) Dot plots and statistics analysis of apoptosis of neutrophils treated with amino acid biosynthesis inhibitors (PHGDH, ASNS and PLA2G3) as indicated. (C) Tumor burdens in NOD/SCID mice. (D) Neutrophil infiltration in tumors determined by CD66b IHC in each group. The red arrow refers to neutrophils. The data represent three independent experiments. Data are presented as the mean ± SD. *P < 0.05; **P < 0.01; ***P< 0.001.
